# The relationship between socioeconomic status and childhood overweight/obesity is linked through paternal obesity and dietary intake: a cross-sectional study in Chongqing, China

**DOI:** 10.1186/s12199-021-00973-x

**Published:** 2021-05-04

**Authors:** Jingyu Chen, Shunqing Luo, Xiaohua Liang, Yetao Luo, Rina Li

**Affiliations:** 1 Department of Ultrasound, Children’s Hospital, Chongqing Medical University, Chongqing, People’s Republic of China; 2 Department of Pediatric General Medicine, Children’s Hospital of Chongqing Medical University, Jinyu Avenue No.20, Yubei, Chongqing, 400010 China; 3 Clinical Epidemiology and Biostatistics Department, Children’s Hospital of Chongqing Medical University, Ministry of Education Key Laboratory of Child Development and Disorders, National Clinical Research Center for Child Health and Disorders Key Laboratory of Pediatrics in Chongqing, China International Science and Technology Cooperation Center of Child Development and Critical Disorders, Chongqing, 400016 China

**Keywords:** Childhood overweight and obesity, Prevalence, Socioeconomic status, Path analyses, Dietary intake

## Abstract

**Background:**

The prevalence of obesity and overweight in childhood has increased dramatically over the past decades globally. Thus, the risk factors of overweight and obesity in children and adolescents must be studied.

**Objectives:**

This study aimed to reveal the prevalence of childhood obesity and examine the relationship between socioeconomic status (SES) and z-body mass index (z-BMI) via parental obesity and dietary intake using path analysis.

**Methods:**

Stratified cluster sampling was used to select 17,007 participants aged 6–12 years on two avenues per region in urban, suburban, and rural areas. Path analysis was conducted to examine the mediators between SES and z-BMI.

**Results:**

The prevalence rates of overweight and obesity were 13.36% and 8.60%, respectively, and were positively correlated with the father’s education level, family income, a birth weight > 3000*g*, a parental obesity history, vegetable intake and red meat intake (all *P* < 0.05). Four mediators (paternal obesity history, red meat intake, vegetable intake, and nutritional supplements) were observed, and the four path analyses were significant (all *P* < 0.05). The adjusted total effects on z-BMI were significant for income (β_Tot_ = 0.03; *P* < 0.01), father’s education (β_Tot_ = 0.05; *P* < 0.001), and region (β_Tot_ = 0.11; *P*<0.001), and the total mediation effects were 20.69%, 16.67%, and 5.36%, respectively. All the variables accounted for 12.60% of the z-BMI variance.

**Conclusions:**

The prevalence of overweight/obesity in children was notable, and the relationship between SES and z-BMI was mediated by paternal obesity history and dietary intake.

**Supplementary Information:**

The online version contains supplementary material available at 10.1186/s12199-021-00973-x.

## Background

The prevalence of overweight and obesity in childhood has increased dramatically over the past decades globally [1]. The prevalence of overweight or obesity in children aged 5–19 years ranged from 6.8~58.3%, and more than 381 million children had overweight/obesity in 2016 worldwide [2]. The prevalence rates of overweight and obesity were 11.7% and 6.8%, respectively, in 2011–2015 among children and adolescents in China [3]; the prevalence continues to increase, particularly in urban areas, and is accompanied by an increase in family income and changes in modern lifestyles [3]. A long-term cohort study found that obesity is the risk factor most strongly associated with a large carotid intima media thickness (cIMT) and an increased pulse wave velocity (PWV) in young adulthood [4, 5], and a large cIMT is an index of an increased risk of cardiovascular disease (CVD) in adulthood. Additionally, children and adolescents with obesity may have psychiatric disorders [6], which may increase the risk of suicide and antisocial behavior in adolescents and adulthood. Therefore, research on the etiology and risk factors for childhood obesity is essential. However, the etiology of childhood overweight/obesity is complex, and its high prevalence may be caused by hereditary factors [7], behavioral factors [8], and environmental factors [9]. Children and adolescents with overweight/obesity may be impacted by unhealthy eating patterns and diet composition [10, 11], adverse perinatal variables (such as maternal obesity [12], overweight at birth [13] and partial breastfeeding [14]) and low socioeconomic status (SES) (including low family income and a low father’s education level) [15]. More specifically, the mechanisms explaining the relationship between SES and overweight/obesity are important for both researchers and healthcare providers and may provide a basis for fostering effective intervention measures. Chung-Ying Lin et al. found that the presence of an eating disorder had a significant mediating effect between psychological distress and excess weight [16]. However, we hypothesize that dietary composition, parental obesity, and birth weight not only have direct effects on obesity but also mediate the association between SES and overweight/obesity among Chinese children.

Most previous studies were based on early surveys and analyses of the factors directly impacting obesity in only some special domains; the current literature does not illustrate the mediating effects between SES and overweight/obesity. In this study, our objectives were to (1) provide prevalence estimates of obesity and overweight in urban-rural children and adolescents in Southwest China in 2014 and perform detailed analyses of prevalence by age, sex, living area, prenatal status, SES and parental obesity; (2) evaluate the multiple variables (including SES, dietary intake, and demographic characteristics) directly impacting childhood overweight/obesity; and (3) examine the indirect effects of SES on overweight/obesity.

## Methods

Two-stage (county and community) stratified cluster sampling was performed to select the participants. The details of participant recruitment and questionnaire collection have been described previously [17]. A cross-sectional study was designed to assess risk factors for overweight/obesity, with evaluations conducted in 2014 from April to September. The inclusion criteria were as follows: (1) participants aged 6~12 years in 2014 [18]; (2) participants who lived in the survey area for more than 6 months [19]; (3) participants with no serious diseases such as nephropathy, CVD, or cancer; and (4) participants for whom informed consent of both the parents and children was provided [20]. This study aimed to include 20 000 subjects, and a high participation rate was obtained, with 90.27% of the contacted children agreeing to participate (18 054); ultimately, 17,007 subjects were included in this study (Supplementary Figure 1). The Institutional Review Board of Children’s Hospital of Chongqing Medical University approved this study, and written informed consent was provided by all the subjects and parents. All the work was conducted under the ethical guidelines of the 1964 Declaration of Helsinki and its later amendments.

### Demographic variables and dietary intake

Demographic information, including age, sex, and geographical area, was collected. SES variables included parents’ occupation, fathers’ education level, household income, single-parent family status, family structure, living with grandparents, and medical insurance. Parental education level was measured on a 4-point scale, ranging from less than high school to postgraduate education. Prenatal variables included birth weight, breastfeeding, and maternal pregnancy-induced hypertension. A family history of obesity was also investigated.

The data regarding dietary intake were collected using a quantitative food frequency questionnaire as described in detail previously [21, 22]. Food was divided into 15 categories, and the total amount of food was divided by the number of days to calculate the average daily intake. Additionally, the proportions of 15 food categories were calculated as the individual food category divided by the total amount of the daily food intake. Nutritional supplements included complex drinks comprising amino acids, unsaturated fatty acids, vitamins (vitamin A and vitamin D) and minerals (e.g., calcium and iron), such as the compound lysine hydrochloride and zinc gluconate granules, probiotics, and phosphatidyl-serine.

### Physical examination

Details of the physical examination have been described previously [23]. The mean arterial pressure (MAP) was calculated as 1/3 systolic blood pressure plus 2/3 diastolic blood pressure [24].

### Diagnostic criteria

A diagnosis of overweight or obesity was based on the sex-specific CDC (Centers for Disease Control and Prevention) BMI-for-age growth charts: overweight was defined as a body mass index (BMI = weight [in kilograms]/height [in meters] ^2^) at or above the 85th percentile and below the 95th percentile, and obesity was defined as a BMI at or above the 95th percentile [25]. Additionally, an SAS code for the 2000 CDC Growth Charts was used to diagnose overweight and obesity [26].

### Mediation model

In this study, five steps were used to construct a mediation effect model. First, this study is based on the hypothesis that socioeconomic indicators will impact childhood obesity by influencing parental obesity and the dietary intake of children (Supplementary Figure 2). Second, SES (income (1 = ~ 500, 2 = ~ 1000, 3 = ~ 2000, 4 = > 2000 RMB/month) and parental education level (1 = ~ 9, 2 = ~ 12, 3 = ~ 15, 4 = > 15 years) and region (0 = rural, 1 = urban/suburban) were considered independent variables, and dietary intake (the proportion of red meat, vegetables, fish, eggs, milk, nuts, pickle, and nutritional supplements) and parental obesity history (mother with obesity (0 = no, 1 = yes) and father with obesity (0 = no, 1 = yes)) were mediator variables; and the z score of the BMI (z-BMI) was the dependent variable based on the mediation model’s hypothesis. Third, the mediator variables were included in the mediation model, if they met the following criteria, with the indexes of the ratio of the root mean square error of approximation (RMSEA) < 0.05, Tucker–Lewis index (TLI) ≥ 0.90, goodness-of-fit index (GFI) ≥ 0.90, and confirmatory fit index (CFI) ≥ 0.90. Additionally, the proportions of red meat, vegetables, nutritional supplements, and father’s obesity were included as mediator variables. Fourth, a multiple mediation model was constructed to test the total mediation effect in the path analysis. Age, sex, birth weight, breast feeding, MAP, and the heart rate were controlled in all models. Finally, the bootstrapping method with 5000 iterations was used to examine the significance of the direct effect, indirect effect and total effect. The bias-corrected percentile method was used to estimate 95% confidence intervals (CI). The indirect effect (β_Ind=_β_1_×β_2_) was calculated as indirect effect 1 (β_1_) multiplied by indirect effect 2 (β_2_). The total effect (β_Tot=_β_Ind+_β_Dir_) was calculated as the indirect effect (β_Ind_) plus the direct effect (β_Dir_). The mediation effect proportion (%) was calculated as (β_Ind_/β_Tot_) ×100%.

### Statistical analyses

Normal distribution continuous variables were reported as means ± standard deviation, and one-way ANOVA was used to calculate the differences among groups of BMI normal, overweight and obesity. Skewed distribution continuous variables of dietary intake were expressed as medians (interquartile range), and the Kruskal-Wallis test was used for comparison among the three groups. The prevalence rates of overweight and obesity by categorical variables were reported as numbers (*n*), percentages of the total (%) and 95% confidence interval (CI), and chi-squared test was used to test the difference among the three groups. Moreover, univariate logistic regression was performed to test the relevance among overweight/obesity and SES, perinatal measures, anthropometric measures, and dietary intake after adjustment by age and gender. Next, all the above significant variables in each domain were added to the multiple logistic regression model to estimate their effects on the prevalence of overweight/obesity. Finally, all the significant variables in each domain model were entered simultaneously to form a full model. The samples with missing data were not included in the analyses. Statistical analysis was performed using SAS 9.4 software (Copyright ©2016; SAS Institute Inc., Cary, NC, USA).

The mediation model was conducted using Amos 24.0, and a *P* value < 0.05 was considered significant.

## Results

### General characteristics

The demographic characteristics of the subjects are presented in Table 1. The mean age was 9.20 ± 1.75 years, and 52.22% (8881/17,007) were male. Significant differences were found in age, BMI, height, weight, waist circumference, heart rate, SBP, DBP, and MAP among the normal, overweight, and obesity groups (all *P* < 0.05). Additionally, significant differences were found in the region, father’s education level, parental occupation, income, living with grandparents, birth weight, and parental obesity among the normal, overweight, and obesity groups (all *P* < 0.05). The proportions of the average intake of vegetables, red meat, fish, eggs, milk, nuts, pickled foods, and nutritional supplements were significantly different among the normal, overweight, and obesity groups (all *P* < 0.05) (Supplementary Table 1).

**Table 1:** General characteristics of children.

Variables	Total	Normal (*n* = 13,272)	Overweight (*n* = 2272)	Obesity (*n* = 1463)	*P*
Anthropometric measures					
Gender, male (*n* (%))	8881 (52.22%)	6368 (47.98%)	1418 (62.41%)	1095 (74.85%)	< 0.001
Age, year	9.20 ± 1.74	9.20 ± 1.75	9.30 ± 1.74	9.05 ± 1.71	< 0.001
BMI, kg/m^2^	17.37 ± 3.08	16.18 ± 1.67	20.22 ± 1.71	23.73 ± 3.83	< 0.001
Height, cm	134.42 ± 11.43	133.54 ± 11.24	137.41 ± 11.67	137.73 ± 11.34	< 0.001
Weight, kg	31.98 ± 9.49	29.30 ± 7.10	38.88 ± 9.39	45.62 ± 11.37	< 0.001
Waist circumference, cm	58.29 ± 8.44	55.49 ± 5.77	65.36 ± 7.32	72.74 ± 9.28	< 0.001
Heart rate, n/min	94.73 ± 12.56	94.54 ± 12.53	94.76 ± 12.59	96.36 ± 12.76	< 0.001
SBP, mmHg	102.97 ± 9.86	101.51 ± 9.30	106.86 ± 9.74	110.15 ± 10.25	< 0.001
DBP, mmHg	63.11 ± 7.62	62.49 ± 7.42	64.54 ± 7.74	66.51 ± 8.03	< 0.001
MAP, mmHg	89.68 ± 8.47	88.50 ± 8.05	92.76 ± 8.34	95.6 ± 8.77	< 0.001
Socioeconomic					
Region					
Urban or suburb	9405 (55.30%)	7100 (53.50%)	1412 (62.15%)	893 (61.04%)	< 0.001
Rural	7602 (44.70%)	6172 (46.50%)	860 (37.85%)	570 (38.96%)	
Father’s education, level, year					
~ 9	8177(49.16%)	6592(50.79%)	973(43.65%)	612(42.89%)	<0.001
~ 12	6393(38.43%)	4851(37.38%)	937(42.04%)	605(42.40%)	
~ 15	1955(11.75%)	1458(11.23%)	300(13.46%)	197(13.81%)	
> 15	109(0.66%)	77(0.59%)	19(0.85%)	13(0.91%)	
Father’s occupation					
Manager	1334 (8.00%)	1011 (7.77%)	206 (9.20%)	117 (8.17%)	< 0.001
Worker	5315 (31.86%)	4052 (31.14%)	773 (34.54%)	490 (34.22%)	
Technicist/Researcher	711 (4.26%)	536 (4.12%)	108 (4.83%)	67 (4.68%)	
Farmer	5410 (32.43%)	4354 (33.46%)	646 (28.87%)	410 (28.63%)	
Others	3914 (23.46%)	3061 (23.52%)	505 (22.56%)	348 (24.30%)	
Mother’s occupation					
Manager	892 (5.35%)	683 (5.25%)	126 (5.63%)	83(5.83%)	< 0.001
Worker	4713 (28.25%)	3615 (27.76%)	700 (31.26%)	398 (27.97%)	
Technicist/Researcher	299 (1.79%)	211 (1.62%)	52 (2.32%)	36 (2.53%)	
Farmer	5119 (30.68%)	3981 (30.57%)	680 (30.37%)	458 (32.19%)	
Others	5660 (33.93%)	4531 (34.80%)	681 (30.42%)	448 (31.48%)	
Income, RMB					
~ 500	843 (5.56%)	719 (6.07%)	71 (3.54%)	53 (4.03%)	< 0.001
~ 1000	1631 (10.76%)	1328 (11.21%)	186 (9.29%)	117 (8.90%)	
~ 2000	2906 (19.17%)	2319 (19.58%)	355 (17.72%)	232 (17.66%)	
> 2000	9782 (64.52%)	7479 (63.14%)	1391 (69.45%)	912 (69.41%)	
Live with grandparents					
No	12,905 (84.61%)	10,073 (84.48%)	1705 (84.91%)	1127 (85.25%)	0.705
Yes	2348 (15.39%)	1850 (15.52%)	303 (15.09%)	195 (14.75%)	
People live with child					
1	730 (4.86%)	574 (4.89%)	88 (4.45%)	68 (5.25%)	0.018
2~3	8053 (53.60%)	6216 (52.91%)	1109 (56.04%)	728 (56.17%)	
4	6240 (41.54%)	4958 (42.20%)	782 (39.51%)	500 (38.58%)	
Medical insurance					
No	12,767 (84.10%)	9956 (83.92%)	1699 (84.91%)	1112 (84.50%)	0.495
Yes	2413 (15.90%)	1907 (16.08%)	302 (15.09%)	204 (15.50%)	
Dietary intakes, %					
Cereals and potatoes	13.49 (7.60, 20.18)	13.42 (7.57, 20.08)	13.70 (7.90, 20.49)	13.60 (7.64, 21.13)	0.457
Vegetables	13.62 (8.47, 20.34)	13.38 (8.34, 20.06)	14.20 (8.82, 20.92)	15.13 (9.44, 21.85)	< 0.001
Fruit	11.25 (6.23, 17.4)	11.34 (6.31, 17.50)	11.18 (6.09, 17.27)	10.55 (5.82, 16.97)	0.070
Red meat	7.10 (4.08,10.72)	6.94 (3.95, 10.55)	7.62 (4.55, 11.29)	7.78 (4.72, 11.43)	< 0.001
Poultry	2.38 (1.23,4.74)	2.39 (1.24, 4.75)	2.32 (1.15,4.56)	2.37 (1.24, 4.77)	0.262
Fish	1.32 (0.49,2.86)	1.34 (0.50, 2.88)	1.26 (0.41, 2.88)	1.22 (0.46, 2.60)	0.013
Eggs	3.66 (1.71,6.31)	3.63 (1.70, 6.27)	3.88 (1.82, 6.70)	3.54 (1.59, 6.11)	0.012
Milk	18.29 (11.63, 26.41)	18.56 (11.79, 26.66)	17.69 (11.22, 25.43)	17.14 (10.86, 25.04)	< 0.001
Bean food	2.22 (1.03, 4.66)	2.24 (1.05, 4.66)	2.23 (1.00, 4.70)	2.09 (0.97, 4.47)	0.188
Nuts	0.95 (0.26, 2.37)	0.97 (0.27, 2.42)	0.87 (0.25, 2.28)	0.84 (0.20, 2.05)	0.001
Mushrooms and algae food	0.85 (0.23, 2.11)	0.84 (0.23, 2.10)	0.89 (0.24, 2.18)	0.88 (0.23, 2.12)	0.522
Oils	4.64 (2.58, 7.43)	4.64 (2.56, 7.43)	4.61 (2.61, 7.46)	4.62 (2.74, 7.43)	0.655
Pickle	0.46 (0.06, 1.33)	0.47 (0.07, 1.34)	0.39 (0.03, 1.30)	0.38 (0.03, 1.37)	0.006
Nutritional supplements	0.00 (0.00, 0.34)	0.00 (0.00, 0.45)	0.00 (0.00, 0.17)	0.00 (0.00, 0.06)	< 0.001
Beverage	1.00 (0.07, 3.51)	1.01 (0.07, 3.52)	0.92 (0.07, 3.21)	1.07 (0.11, 3.83)	0.082
Perinatal measures					
Gestational hypertension					
No	14,557 (98.49%)	11,382 (98.55%)	1922 (98.16%)	1253 (98.51%)	0.435
Yes	223 (1.51%)	168 (1.45%)	36 (1.84%)	19 (1.49%)	
Birth weight, g					
~ 3000	4138 (27.19%)	3405 (28.63%)	461 (22.98%)	272 (20.61%)	< 0.001
~ 3600	6619 (43.49%)	5111 (42.97%)	914 (45.56%)	594 (45.00%)	
> 3600	4464 (29.33%)	3379 (28.41%)	631 (31.46%)	454 (34.39%)	
Breast feeding, month					
0~3	3751 (25.00%)	2890 (24.66%)	507 (25.64%)	354 (27.13%)	0.152
4~10	7408 (49.38%)	5789 (49.39%)	994 (50.28%)	625 (47.89%)	
> 10	3844 (25.62%)	3042 (25.95%)	476 (24.08%)	326 (24.98%)	
Father with obesity					
No	12,662 (83.93%)	10,152 (86.06%)	1562 (78.97%)	948 (72.26%)	< 0.001
Yes	2425 (16.07%)	1645 (13.94%)	416 (21.03%)	364 (27.74%)	
Mother with obesity					
No	13,592 (90.13%)	10,801 (91.62%)	1709 (86.18%)	1082 (82.66%)	< 0.001
Yes	1489 (9.87%)	988 (8.38%)	274 (13.82%)	227 (17.34%)	

*BMI* body mass index, *DBP* diastolic blood pressure, *SBP* systolic blood pressure, *MAP* mean arterial pressure

### Prevalence of overweight and obesity

Table 2 displays the results of the prevalence of childhood overweight and obesity: the prevalence rates of overweight and obesity were 13.36% and 8.60%, respectively. The prevalence of overweight and obesity was higher in children with the characteristics of male sex, living in urban or suburban areas, and a history parental obesity than in their counterparts (all *P* < 0.05). The father’s education level, income, and birth weight were positively correlated with the prevalence of overweight and obesity (all *P* for trend < 0.05). Additionally, the greater was the number of family members living with children, the lower was the prevalence of obesity in children (*P* for trend < 0.05). Finally, the prevalence rates of overweight and obesity were significantly different according to parental occupation (all *P* < 0.05). The hierarchical analysis for the prevalence of overweight and obesity by sex and urban/suburban-rural areas are displayed in Supplementary Tables 2 and 3, and the male adolescents living in an urban area had the highest prevalence of overweight (17.99%) and obesity (13.76%) than other counterparts.

**Table 2:** Prevalence of overweight/obesity of children aged 6–12 years.

Variables	*N*	Overweight			Obesity		
		Prevalence (95%CI)	*P*	P for trend	Prevalence (95%CI)	*P*	P for trend
Total		13.36% (12.85%, 13.88%)			8.60% (8.19%, 9.03%)		
Anthropometric measures							
Gender							
Male	8881 (52.22%)	15.97% (15.21%, 16.75%)	< 0.001	–	12.33% (11.65%, 13.03%)	< 0.001	–
Female	8126 (47.78%)	10.51% (9.85%, 11.20%)			4.53% (4.09%, 5.00%)		
Age, year							
6	2046(12.03%)	11.83% (10.46%, 13.31%)	0.047	0.002	9.78% (8.52%, 11.14%)	0.039	0.001
7	3153 (18.54%)	12.34% (11.21%, 13.54%)			9.01% (8.03%, 10.06%)		
8	2876 (16.91%)	13.14% (11.93%, 14.43%)			8.90% (7.89%, 10.00%)		
9	2689 (15.81%)	14.35% (13.05%, 15.74%)			8.44% (7.42%, 9.56%)		
10	2770 (16.29%)	13.65% (12.39%, 14.98%)			8.52% (7.51%, 9.62%)		
11	2669 (15.69%)	14.46% (13.15%, 15.85%)			7.94% (6.94%, 9.03%)		
12	804 (4.73%)	14.05% (11.73%, 16.65%)			5.97% (4.43%, 7.84%)		
Socioeconomic index							
Region							
Urban or suburb	9405 (55.30%)	15.01% (14.30%, 15.75%)	< 0.001	–	9.49% (8.91%, 10.11%)	< 0.001	–
Rural	7602 (44.70%)	11.31% (10.61%, 12.05%)			7.50% (6.92%, 8.11%)		
Father’s education, level, year^a^							
~9	8177 (49.16%)	11.90% (11.21%, 12.62%)	< 0.001	< 0.001	7.48% (6.92%, 8.08%)	< 0.001	< 0.001
~12	6393 (38.43%)	14.66% (13.80%, 15.55%)			9.46% (8.76%, 10.21%)		
~15	1955 (11.75%)	15.35% (13.77%, 17.02%)			10.08% (8.78%, 11.50%)		
>15	109 (0.66%)	17.43% (10.83%, 25.87%)			11.93% (6.51%, 19.53%)		
Father’s occupation^b^							
Manager	1334 (8.00%)	15.44% (13.54%, 17.49%)	< 0.001	–	8.77% (7.31%, 10.42%)	0.028	–
Worker	5315 (31.86%)	14.54% (13.61%, 15.52%)			9.22% (8.45%, 10.03%)		
Technicist/Researcher	711 (4.26%)	15.19% (12.63%, 18.04%)			9.42% (7.38%, 11.81%)		
Farmer	5410 (32.43%)	11.94% (11.09%, 12.83%)			7.58% (6.89%, 8.32%)		
Others	3914 (23.46%)	12.90% (11.87%, 13.99%)			8.89% (8.02%, 9.83%)		
Mother’s occupation^c^							
Manager	892 (5.35%)	14.13% (11.91%, 16.59%)	< 0.001	–	9.30% (7.48%, 11.40%)	0.053	–
Worker	4713 (28.25%)	14.85% (13.85%, 15.90%)			8.44% (7.67%, 9.28%)		
Technicist/Researcher	299 (1.79%)	17.39% (13.27%, 22.17%)			12.04% (8.58%, 16.28%)		
Farmer	5119 (30.68%)	13.28% (12.37%, 14.24%)			8.95% (8.18%, 9.76%)		
Others	5660 (33.93%)	12.03% (11.20%, 12.91%)			7.92% (7.22%, 8.65%)		
Income, RMB^d^							
~ 500	843 (5.56%)	8.42% (6.64%, 10.51%)	< 0.001	< 0.001	6.29% (4.74%, 8.14%)	< 0.001	< 0.001
~ 1000	1631 (10.76%)	11.40% (9.90%, 13.05%)			7.17% (5.97%, 8.54%)		
~ 2000	2906 (19.17%)	12.22% (11.05%, 13.46%)			7.98% (7.02%, 9.03%)		
> 2000	9782 (64.52%)	14.22% (13.53%, 14.93%)			9.32% (8.75%, 9.92%)		
Live with grandparents^e^							
No	12,905 (84.61%)	13.21% (12.63%, 13.81%)	0.685	–	8.73% (8.25%, 9.23%)	0.498	–
Yes	2348 (15.39%)	12.90% (11.57%, 14.33%)			8.30% (7.22%, 9.50%)		
People live with child^f^							
1	730 (4.86%)	12.05% (9.78%, 14.64%)	0.062	0.181	9.32% (7.31%, 11.66%)	0.075	0.028
2~3	8053 (53.60%)	13.77% (13.03%, 14.54%)			9.04% (8.42%, 9.69%)		
4	6240 (41.54%)	12.53% (11.72%, 13.38%)			8.01% (7.35%, 8.71%)		
Medical insurance^g^							
No	12,767 (84.10%)	13.31% (12.72%, 13.91%)	0.291	–	8.71% (8.23%, 9.21%)	0.682	–
Yes	2413 (15.90%)	12.52% (11.22%, 13.90%)			8.45% (7.37%, 9.64%)		
Perinatal measures							
Gestational hypertension^h^							
No	14557 (98.49%)	13.20% (12.66%, 13.76%)	0.199	–	8.61% (8.16%, 9.07%)	0.963	–
Yes	223 (1.51%)	16.14% (11.57%, 21.64%)			8.52% (5.21%, 12.99%)		
Birth weight, g^i^							
~3000	4138 (27.19%)	11.14% (10.20%, 12.14%)	< 0.001	< 0.001	6.57% (5.84%, 7.37%)	< 0.001	< 0.001
~3600	6619 (43.49%)	13.81% (12.99%, 14.66%)			8.97% (8.30%, 9.69%)		
>3600	4464 (29.33%)	14.14% (13.13%, 15.19%)			10.17% (9.30%, 11.09%)		
Breast feeding, month^j^							
0~3	3751 (25.00%)	13.52% (12.44%, 14.65%)	0.238	0.142	9.44% (8.52%, 10.42%)	0.178	0.142
4~10	7408 (49.38%)	13.42% (12.65%, 14.22%)			8.44% (7.81%, 9.09%)		
>10	3844 (25.62%)	12.38% (11.36%, 13.47%)			8.48% (7.62%, 9.41%)		
Father with obesity^k^							
No	12,662 (83.93%)	12.34% (11.77%, 12.92%)	< 0.001	–	7.49% (7.03%, 7.96%)	< 0.001	–
Yes	2425 (16.07%)	17.15% (15.67%, 18.72%)			15.01% (13.61%, 16.49%)		
Mother with obesity^l^							
No	13,592 (90.13%)	12.57% (12.02%, 13.14%)	< 0.001	–	7.96% (7.51%, 8.43%)	< 0.001	–
Yes	1489 (9.87%)	18.40% (16.46%, 20.47%)			15.25% (13.46%, 17.17%)		

^a^A total of 373 subjects having missing values in mother’s education level.

^b^A total of 323 subjects having missing value in father’s occupation.

^c^A total of 324 subjects having missing value in mother’s occupation.

^d^A total of 1845subjects having missing values in parents’ income.

^e^A total of 1754subjects having missing values in live with grandparents.

^f^A total of 1984 subjects having missing values in people live with.

^g^A total of 1827 subjects having missing values in medical insurance.

^g^A total of 1827 subjects having missing values in medical insurance.

^i^A total of 1786 subjects having missing values in birth weight.

^j^A total of 2004 subjects having missing values in breastfeeding.

^k^A total of 1920 subjects having missing values in father with obesity.

^l^A total of 1926 subjects having missing values in mother with obesity.

### Risk factors for childhood overweight and obesity

The univariate logistic regression model showed that living in urban or suburban areas, a higher father’s education level and family income, birth weight, and parental obesity and a high proportion of red meat intake were risk factors for childhood overweight and obesity (all *P* < 0.05). However, more than 10 months of breastfeeding, high proportions of milk and nut intake, and nutritional supplements were protective factors against childhood overweight and obesity (all *P* < 0.05) (Supplementary Table 4).

The relationship of SES, perinatal measurements, anthropometric, and dietary intake with childhood overweight and obesity was analyzed by building a multivariable logistic regression model (Supplementary Table 5). In the SES model, living in urban or suburban areas, the fathers’ education level and family income significantly affected overweight and obesity (*P* < 0.05), explaining 1.38% of the variance. In the perinatal measurement model, birth weight, and parental obesity were risk factors for overweight and obesity in children, but breast feeding was a protective factor against obesity, explaining 2.90% of the variance. Additionally, MAP was a risk factor for overweight and obesity in children. In the dietary intake model, the proportion of vegetable intake, red meat intake, and nutritional supplements were risk factors for childhood overweight and obesity (all *P* < 0.05), explaining 0.99% of the variance.

### Full model analyzing the risk factors of BP

The full model included the significant variables from the SES, perinatal, anthropometric, and dietary variables models, as shown in Table 3. Living in urban or suburban areas, the father’s education level, birth weight, parental obesity, MAP, and the proportions of vegetable and red meat intake were significantly positively associated with overweight and obesity. Additionally, the proportion of egg intake was a risk factor for overweight, and the proportion of nutritional supplements was a protective factor against obesity. The full model explained 16.18% of the variance in overweight and obesity.

**Table 3:** Full model of risk factors for childhood overweight/obesity

Variables	Overweight vs. Normal			Obesity vs. Normal			*R*^2^
	β^a^	*P*	OR(95%CI)	β^a^	*P*	OR(95%CI)	
General characteristics							
Region (urban or suburb vs. rural)	0.385	< 0.001	1.469 (1.307,1.651)	0.509	< 0.001	1.664 (1.438,1.926)	16.18%
Father education, ref. ≤ 9 years							
~ 12	0.210	0.001	1.234 (1.093,1.393)	0.171	0.027	1.187 (1.020,1.381)	
~ 15	0.227	0.009	1.255 (1.058,1.490)	0.260	0.015	1.296 (1.051,1.598)	
> 15	0.143	0.662	1.154 (0.607,2.196)	0.090	0.824	1.094 (0.496,2.413)	
Income, ref. ≤ 500 RMB							
~ 1000	0.233	0.160	1.263 (0.912,1.749)	0.107	0.602	1.112 (0.745,1.661)	
~ 2000	0.222	0.150	1.249 (0.923,1.690)	0.123	0.513	1.131 (0.782,1.636)	
> 2000	0.314	0.033	1.368 (1.026,1.825)	0.215	0.228	1.240 (0.874,1.759)	
Perinatal measures							
Birth weight, ref. ~ 3000 g							
3000~3600	0.213	0.002	1.237 (1.082,1.415)	0.312	< 0.001	1.366 (1.149,1.624)	
> 3600	0.284	< 0.001	1.329 (1.148,1.539)	0.435	< 0.001	1.545 (1.283,1.860)	
Breast feeding, ref. 0~3 month							
4~10	0.037	0.578	1.038 (0.911,1.182)	− 0.081	0.321	0.922 (0.787,1.082)	
> 10	− 0.002	0.976	0.998 (0.854,1.166)	− 0.119	0.219	0.887 (0.734,1.074)	
Father with obesity (Yes vs. No)	0.364	< 0.001	1.439 (1.255,1.650)	0.719	< 0.001	2.053 (1.756,2.399)	
Mother with obesity (Yes vs. No)	0.505	< 0.001	1.657 (1.405,1.955)	0.798	< 0.001	2.221 (1.841,2.679)	
Anthropometric measures							
MAP, mmHg	0.064	< 0.001	1.066 (1.058,1.074)	0.109	< 0.001	1.115 (1.105,1.125)	
Dietary intaking							
Vegetables, %	0.008	0.007	1.008 (1.002,1.014)	0.016	< 0.001	1.016 (1.009,1.023)	
Red meat, %	0.013	0.006	1.013 (1.004,1.022)	0.022	< 0.001	1.023 (1.012,1.034)	
Eggs, %	0.020	0.001	1.020 (1.008,1.033)	− 0.006	0.480	0.994 (0.977,1.011)	
Nutritional supplements, %	− 0.019	0.062	0.981 (0.961,1.001)	− 0.029	0.035	0.971 (0.945,0.998)	

*MAP* mean arterial pressure

^a^A total of 12813 subjects being included in full model and adjusted age and gender

### Mediation model

The mediation model is shown in Supplementary Figure 2. The effect of SES on z-BMI with mediators of parental obesity and diet proportion is shown in Table 4. The model fit indexes of all the mediation models are shown in Supplementary Table 6. The mediation effects of paternal obesity, proportion of red meat intake, and proportion of nutritional supplements on the correlations between income and z-BMI were estimated at 8.70%, 6.15%, and 2.90%, with significant indirect effects of 0.006, 0.004, and 0.003 (Supplementary Figure 3 a–c and Table 4), respectively, after adjustment for age, sex, birth weight, breast feeding, MAP, and the heart rate.

**Table 4:** The effect of SES on the z-score of body mass index (z-BMI) with mediators of parental obesity and the proportion of diet

Path	Indirect effect			Direct effect (βDir)	Total effect (β_Tot_)	Mediation effect (%)
	β_1_	β_2_	β_Ind_			
Income						
Income→Mother with obesity→z-BMI	− 0.028**	0.100***	− 0.003**	0.071 (0.055, 0.088)***	0.068 (0.052, 0.085)***	4.41
Income→Father with obesity→z-BMI	0.049***	0.115***	0.006***	0.063 (0.047, 0.080)***	0.069 (0.053, 0.086)***	8.70
Income→The proportion of vegetables→z-BMI	− 0.003	0.045***	− 0.0001	0.068 (0.052, 0.085)***	0.068 (0.052, 0.085)***	0.15
Income→The proportion of red meat→z-BMI	0.068***	0.057***	0.004***	0.065 (0.049, 0.082)***	0.069 (0.053, 0.086)***	6.15
Income→The proportion of nutritional supplements→z-BMI	− 0.055***	− 0.040***	0.002***	0.067 (0.050, 0.084)***	0.069 (0.052, 0.086)***	2.90
Father education						
Father education→Mother with obesity→z-BMI	− 0.023*	0.100***	− 0.002*	0.077 (0.061, 0.094)***	0.075 (0.059, 0.091)***	2.67
Father education→Father with obesity→z-BMI	0.048***	0.115***	0.006***	0.072 (0.055, 0.088)***	0.077 (0.061, 0.093)***	7.79
Father education→The proportion of vegetables→z-BMI	0.012	0.044***	0.001	0.076(0.060, 0.093)***	0.077 (0.061, 0.094)***	1.30
Father education→The proportion of red meat→z-BMI	0.065***	0.056***	0.004***	0.073 (0.057, 0.090)***	0.077 (0.061. 0.094)***	5.19
Father education→The proportion of nutritional supplements→z-BMI	− 0.077***	− 0.038***	0.003***	0.074 (0.057, 0.091)***	0.077 (0.061. 0.094)***	3.90
Region						
Region (Urban or suburban vs. rural)→Mother with obesity→z-BMI	− 0.009	0.099***	− 0.001	0.124(0.108, 0.139)***	0.123(0.108, 0.139)***	0.81
Region (Urban or suburban vs. rural)→Father with obesity→z-BMI	0.023*	0.116***	0.003*	0.124(0.108, 0.140)***	0.127(0.111, 0.143)***	2.36
Region (Urban or suburban vs. rural)→The proportion of vegetables→z-BMI	0.050***	0.037***	0.002***	0.127(0.111, 0.144)***	0.129(0.113, 0.146)***	1.55
Region (Urban or suburban vs. rural)→The proportion of red meat→z-BMI	0.062***	0.053***	0.003***	0.126(0.110, 0.143)***	0.129(0.113, 0.146)***	2.33
Region (Urban or suburban vs. rural)→The proportion of nutritional supplements→z-BMI	− 0.091***	− 0.032***	0.003***	0.126(0.110, 0.143)***	0.129(0.113, 0.146)***	2.33

Age, gender, birth weight, breast feeding, mean arterial pressure and heart rate were controlled in the hypothesized model

β = standardized regression coefficient; β_1_ = indirect effect 1; β_2_ = indirect effect 2; β_Ind_ = total indirect effect; β_Dir_ = direct effect; β_Tot_ = total effect; z-BMI = the z-score of body mass index. *P < 0.05; **P < 0.01; ***P < 0.001

Similarly, the mediation effects of paternal obesity, the proportion of red meat intake, and the proportion of nutritional supplements on the correlation between the father’s education and z-BMI were estimated at 7.79%, 5.19%, and 3.90%, with significant indirect effects of 0.006, 0.004, and 0.003, respectively (Supplementary Figure 3 d–f and Table 4).

The mediation effects of the proportion of red meat intake, vegetable intake, and nutritional supplements on the correlation between region and z-BMI were estimated at 1.55%, 2.33%, and 2.33%, with significant indirect effects of 0.002, 0.003, and 0.003, respectively (Supplementary Figure 4 a–c and Table 4).

### Path analysis of the multiple mediation model

**Fig. 1:**
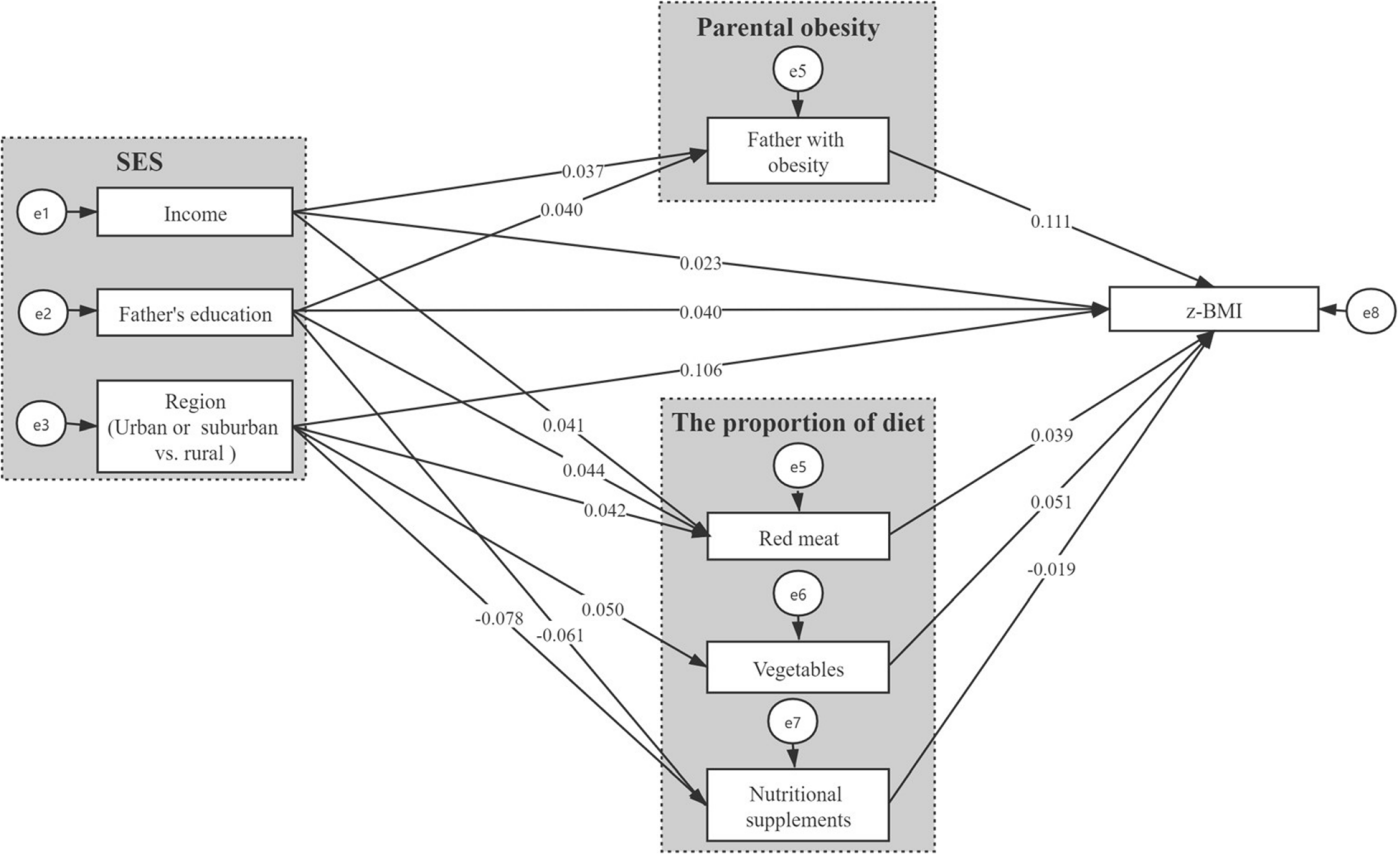
Mediation effect of parental obesity and the proportion of diet on the association between SES and z-BMI in path analysis. (Original to this manuscript). Age, gender, birth weight, breast feeding, the mean arterial pressure and the heart rate were controlled in the hypothesized model. *SES* socioeconomic status, *z*-*BMI* z-score of body mass index. The coefficients are standardized path coefficients. All the path coefficients were significant (*P* < 0.05)

A multiple mediation model with four mediators of SES and z-BMI was established and is shown in Fig. 1. After controlling for age, sex, birth weight, breast feeding, MAP, and the heart rate, the final model showed a good model fit: χ^2^/df = 7.478, GFI = 0.996, CFI = 0.953, TLI = 0.911, and RMSEA = 0.022. The z-BMI was positively related to income, the father’s education level, living in an urban area, paternal obesity, the proportion of vegetable intake, and the proportion of red meat intake but negatively related to the proportion of nutritional supplements (Fig. 1). Table 5 shows the direct, indirect, and total effects of the multiple mediation model in the path analysis. The adjusted total effects on z-BMI were significant for income (β_Tot_ = 0.029, *p* < 0.01), the father’s education level (β_Tot_ = 0.048, *p* < 0.001), and region (β_Tot_ = 0.112, *p* < 0.001). The total mediation effects of income, father’s education level, and region on z-BMI were estimated at 20.69%, 16.67%, and 5.36%, with significant total indirect effects of 0.006, 0.008, and 0.006 (Table 5), respectively. All the variables in the multiple mediation model accounted for 12.60% of the variance in z-BMI.

**Table 5:** Direct, indirect, and total effects of the multiple mediation mode in path analysis

Effect	B (bootstrapping SE)	β	LL	UL
Direct effect				
Income→z-BMI	0.029 (0.011)	0.023*	0.006	0.041
Father education→z-BMI	0.020 (0.005)	0.040***	0.023	0.057
Region(Urban or suburban vs. rural)→z-BMI	0.232 (0.019)	0.106***	0.089	0.124
Indirect effect				
Income→Father with obesity/Birth weight/The proportion of red meat→Overweight/Obesity	0.008 (0.001)	0.006***	0.005	0.011
Father education→Father with obesity/The proportion of red meat/The proportion of nutritional supplements→Overweight/Obesity	0.004 (0.001)	0.008***	0.004	0.009
Region(Urban or suburban vs. rural)→Birth weight/The proportion of red meat/The proportion of vegetables/ The proportion of nutritional supplements→Overweight/Obesity	0.012 (0.002)	0.006***	0.004	0.008
Total effect				
Income→z-BMI	0.037 (0.005)	0.029**	0.012	0.047
Father education→z-BMI	0.024 (0.011)	0.048***	0.031	0.066
Region(Urban or suburban vs. rural)→z-BMI	0.245 (0.019)	0.112***	0.095	0.130

Age, gender, birth weight, breast feeding, mean arterial pressure and heart rate were controlled in the hypothesized model

B = unstandardized path coefficient; SE = standard error; β = standardized path coefficient; LL = lower limit at 95% confidence interval; UL = upper limit at 95% confidence interval; z-BMI = the z-score of body mass index. *P < 0.05; **P < 0.01; ***P < 0.001

## Discussion

To our best knowledge, this study is the first to illustrate the risk factors for childhood overweight/obesity and mediation effects between SES and z-BMI. First, we used a large sample size to identify factors (i.e., SES, birth weight, parental obesity, and dietary composition) impacting the high prevalence of childhood overweight/obesity; second, we used parallel mediation analysis to assess whether the temporal association between SES and overweight/obesity was mediated via different mediators (i.e., dietary composition and parental obesity). The study hypotheses were supported by the parallel mediation analyses using path analysis. The results verified that SES was associated with overweight/obesity through four mediators and that all the mediators were significant.

The prevalence of overweight and obesity in boys in Chongqing was higher than those reported in the China Health and Nutrition Survey (CHNS) and China Nutritional Transition Cohort Study (CNTCS), but the prevalence of obesity in girls in Chongqing was lower than those reported in the CHNS and CNTCS [27]. Because different diagnostic criteria were used, the different SES, lifestyle, and nutrition status may contribute to the difference in obese prevalence. Additionally, we analyzed a larger sample from urban, suburban, and rural areas to illustrate the prevalence in children in Southwest China, resulting in a more reliable conclusion. We found that children with a better SES (living in urban or suburban areas with a higher father’s education level and higher family income) had a higher prevalence of overweight and obesity than those with a lower SES, a finding that was consistent with other studies [28, 29]; however, this result also contrasted with those of other studies [30] and requires further study to verify the current findings. Yang Liu et al. [31] found a significant protective interaction effect between paternal education and household wealth on the obesity risk in girls but no significant difference in the childhood obesity prevalence in children with different household wealth levels, fathers’ education levels, mothers’ education levels, or residence registration areas, which was inconsistent with our results. The different effects of SES on obesity from the aforementioned research may be due to different classification criteria, representation areas and sample sizes. We used detailed classification criteria (4 levels) to define the education level of parents, and representative urban-rural areas were chosen, suggesting that the results of our study might be more reliable than those of previous studies. Furthermore, consistent with previous studies, a high birth weight [32] and parental obesity [33] were positively correlated with childhood obesity in this study.

The family SES variables not only had direct effects on childhood obesity but also impacted childhood obesity through the indirect effects of a history of parental obesity (before pregnancy) and dietary composition. The four mediators in our study help explain why SES is a contributor to childhood obesity. The associations between SES and both parental and childhood obesity are controversial. Prior research [29] revealed a lower SES and educational level increased the risk of adult obesity, while a higher SES was positively correlated with childhood obesity in developed areas. The present study demonstrated that a higher family income and a higher education level are related to an increased risk of parental obesity, and a history of parental obesity contributed to an increased z-BMI, suggesting that children with a high SES (income and father’s education) may gain excess weight because of a history of parental obesity. The mechanisms by which parental obesity could affect an offspring’s BMI may be explained by shared genes and lifestyles [12, 34].

Another mediator related to a high SES (income, father’s education and region)—the proportion of red meat intake—was also a significant mediator in the findings of the current study. Unsurprisingly, parents with a higher SES and education level may allow their children to consume more red meat than those with a lower SES and education level, and the excessive intake of red meat may lead to weight gain [35]. A previous study [36] demonstrated that a lower family income was correlated with less red meat consumption than a higher family income. Therefore, red meat appears to be another significant mediator in the association of SES with obesity.

Regarding vegetable intake, Victoria Miller et al. [37] found that adults in urban areas consumed more vegetables than those in rural areas in lower-middle-income countries such as China, and the consumption of fruits and vegetables is low in low-income populations. In this study, we found that vegetable intake was a significant mediator of the correlations of urban and suburban areas with z-BMI, indicating that children living in urban areas consume more vegetables than their counterparts, and high vegetable consumption was positively correlated with an increased z-BMI. The relationship between vegetable intake and obesity is controversial [38]. Some previous studies [39, 40] found that high vegetable intake was associated with a low BMI, whereas other studies found that vegetable consumption was not significantly related to BMI in children [41, 42]. Consistent with our results, the results from the Young-HUNT study [43] found that less vegetable intake had a lower risk of obesity (OR = 0.6 in girls; OR = 0.8 in boys), but the association was not significant. Additionally, another study from Southern Italy [42] found that vegetable intake was significantly correlated with other dietary intake. Different from other regions, the eating habits of people in Chongqing include fried vegetables and meat together with a large amount of oil, which could be explained by a mass use of cooking oil to prepare vegetables, as Chinese people enjoy sauteed vegetables. Additionally, our study found that vegetable intake was positively correlated with red meat intake (*R* = 0.26; *P* < 0.01) and oil intake (*R* = 0.10; *P* < 0.01), which may partly explain the positive correlation between vegetable intake and obesity in our study.

Furthermore, nutritional supplements were a mediator between SES (father’s education level and urban-rural areas) and z-BMI in the path analysis, indicating that nutritional supplement intake was negatively correlated with SES variables (higher father’s education level and living in urban or suburban areas) and z-BMI. A previous study [44] reported that a low percentage of overweight/obese college students consumed nutritional supplements and that lean students drank more nutritional supplements than overweight/obese students to increase their BMI; our study identified these results in children. However, a higher father’s education level was positively correlated with the consumption of nutritional supplements in a previous study [44], a finding that was inconsistent with ours, suggesting that health education has changed the understanding of health concepts in parents in China.

In this study, all the variables accounted for 12.60% of the z-BMI variance. The variables included in mediation model analyses explained less than the full model in Table 3 (12.60% vs. 16.18%), because the full model included more variables (added birth weight, breast feeding, mother’s obesity, MAP, and intake of eggs) than the variables in the mediation model. Additionally, results from C Maffeis et al. [45] found that the mother’s BMI and TV viewing accounted for 17% of the children’s relative body mass index (rel BMI) variance at the age of 8 years while the parents’ BMIs accounted for 13.5% of the children’s rel BMI variance at the age of 12 years, a finding that was comparable with ours. Another study found that parents’ BMI, dinner intake (percentage of energy intake), an index of energy intake validity, and sex could explain approximately 19% of inter-individual fat mass percentage variability [46], which was higher than our results.

## Limitations

The present study has several limitations. First, the dietary intake data collected in the study were self-reported using a dietary frequency table; thus, recall bias is inevitable. Nevertheless, the use of self-reported dietary intake data has been advocated in biomedical research [17]. Second, the mediation effect of dietary intake in our study might not be generalizable to developed Western countries, given that dietary intake patterns are different among different areas and seasons [46]. Future studies must investigate whether the mediation model supported in the present study can be extended to other countries with different dietary habits in different seasons. Third, we only surveyed the fathers’ education level at this cross-sectional study because a study reported the fathers’ education level significantly affected cardiovascular disease [5] and obesity [47]. Finally, the data of parental obesity were calculated using the self-reported height and weight before pregnancy, which tends to be imprecise. We collected the data twice to reduce recall bias, and the medical records were checked for differences between the two recordings.

## Conclusions

This study provides additional evidence that children with the characteristics of male sex, living in urban or suburban areas, a high father’s education level and family income, high birth weight, and a parental history of obesity have a higher prevalence of overweight and obesity than their counterparts in Southwest China. Additionally, the present study found four important mediators (paternal obesity, red meat intake, vegetable intake, and nutritional supplements) in the temporal association between SES and z-BMI. Healthcare providers may gain insight from such findings and help foster appropriate and effective programs to improve healthy dietary habits, such as the implementation of comprehensive interventions for communities, schools, families, and hospitals to prevent overweight and obesity in children and adolescents. However, the present findings should be interpreted with caution. Specifically, the mediators and weight status were collected simultaneously. Therefore, except for paternal obesity, the causal relationships between the mediators and z-BMI are unclear. Future large-sample-size cohort studies are needed to examine the postulation that programs for improving mediating factors can address obesity problems among children.

## Supplementary Information

**Additional file 1 Fig. S1.** The flow chart of sample included in this study.

**Additional file 2 Fig. S2.** The mediation model in present study.

**Additional file 3 Fig. S3.**

**Additional file 4 Fig. S4.**

**Additional file 5 Table S1.** Dietary intakes.

**Additional file 6 Table S2.** The prevalence of overweight in adolescent by region and sex subgroups.

**Additional file 7 Table S3.** The prevalence of obesity in adolescent by region and sex subgroups.

**Additional file 8 Table S4.** Results of univariate logistic model of the risk factors for childhood overweight/obesity.

**Additional file 9 Table S5.** Model of SES, perinatal and anthropometric for childhood overweight and Obesity.

**Additional file 10 Table S6.** Mediation model fit indexes.

## Data Availability

The datasets supporting the conclusions of this article are available from Xiaohua Liang (contact via xiaohualiang@hospital.cqmu.edu.cn or liangxiaohua666@sina.com).

## Abbreviations

SES: Socioeconomic status
z-BMI: z-Body mass index
cIMT: Carotid intima media thickness
PWV: Pulse wave velocity
CVD: Cardiovascular disease
MAP: Mean arterial pressure
CDC: Centers for Disease Control and Prevention
CI: Confidence interval
RMSEA: Ratio of the root mean square error of approximation
TLI: Tucker–Lewis Index
GFI: Goodness-of-fit index
CFI: Confirmatory fit index
CHNS: China Health and Nutrition Survey
CNTCS: China Nutritional Transition Cohort Study
