# Supplementary figures and images for: The relationship between socioeconomic status and childhood overweight/obesity is linked through paternal obesity and dietary intake: a cross-sectional study in Chongqing, China
Source: Environ Health Prev Med. 2021 May 4;26:56. doi: 10.1186/s12199-021-00973-x (PMC8097861; doi:10.1186/s12199-021-00973-x)

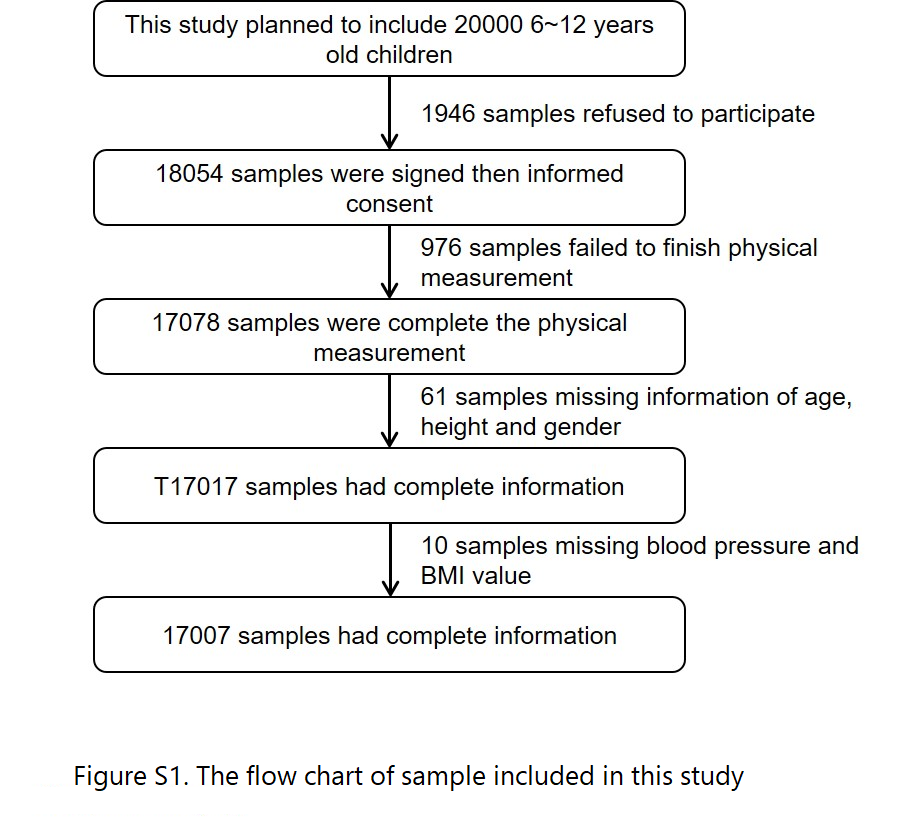

Supplement: Supplementary file 1 — Additional file 1 Fig. S1. The flow chart of sample included in this study. [file 12199_2021_973_MOESM1_ESM.png]

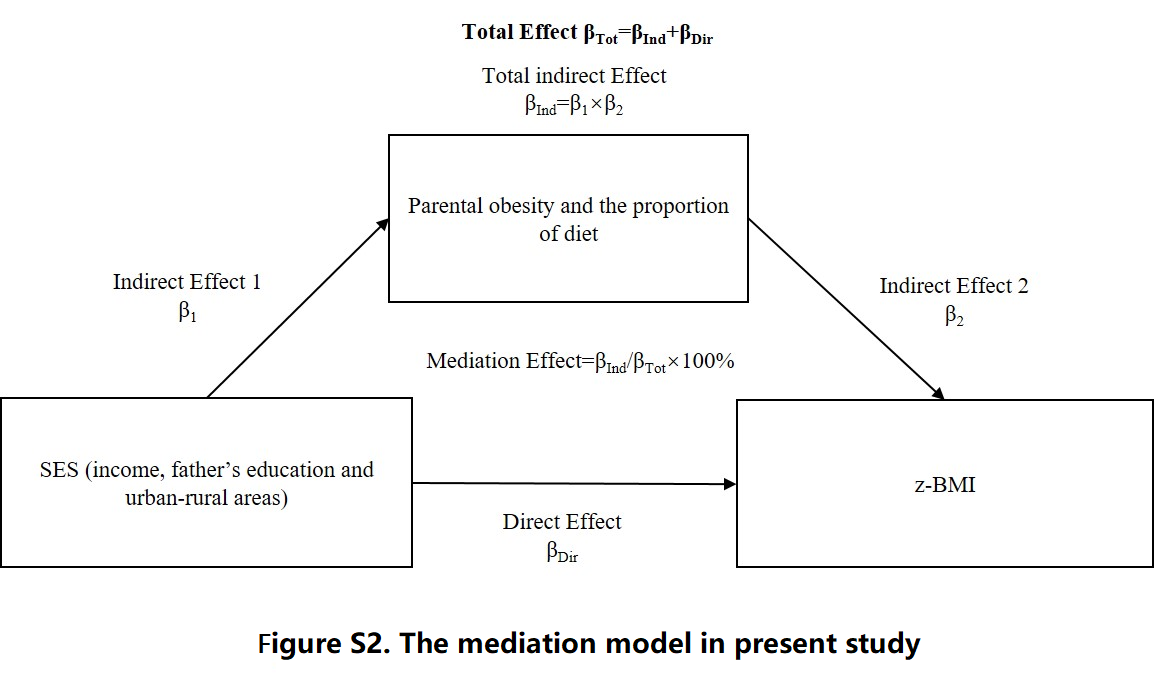

Supplement: Supplementary file 2 — Additional file 2 Fig. S2. The mediation model in present study. [file 12199_2021_973_MOESM2_ESM.png]

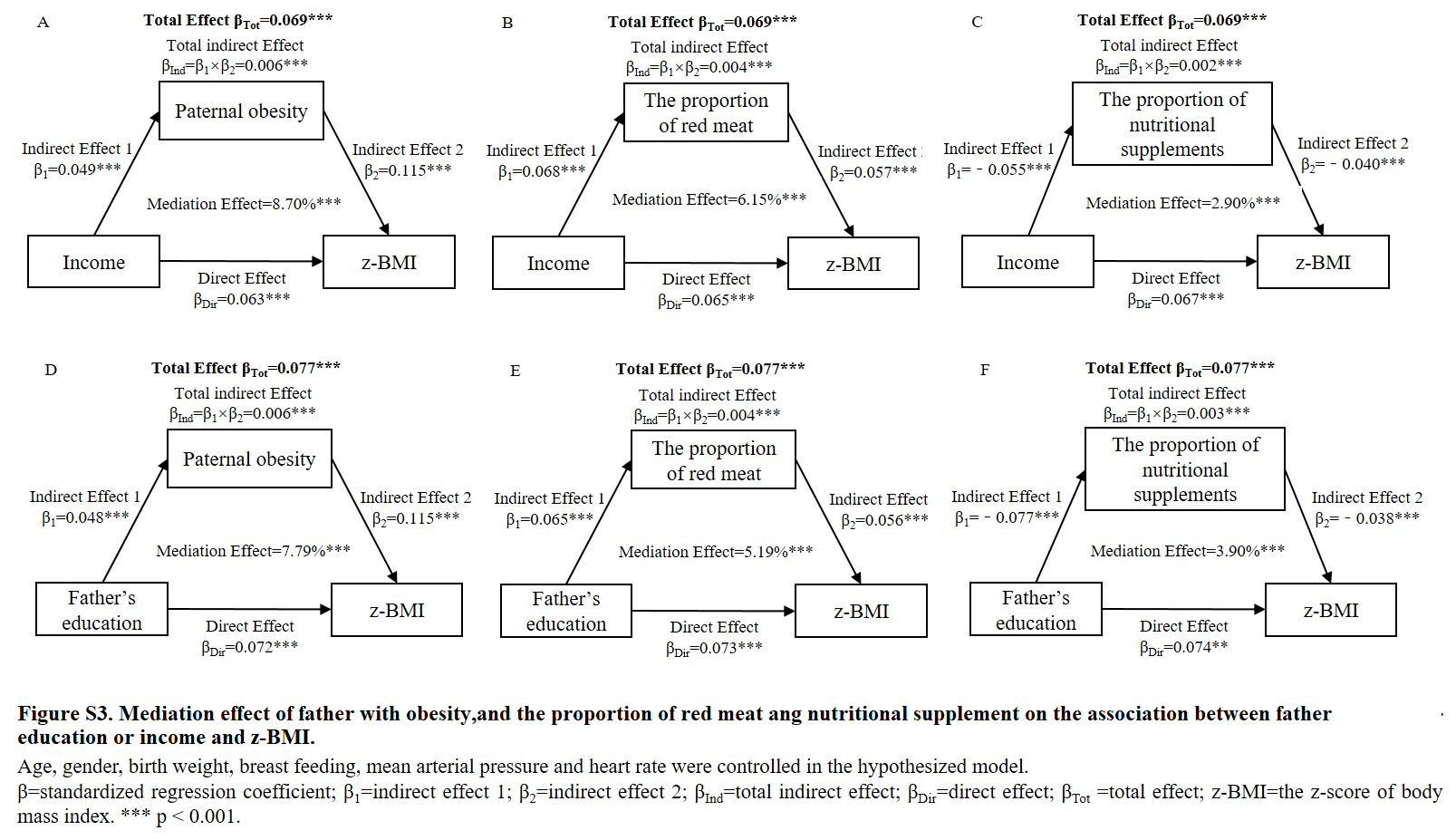

Supplement: Supplementary file 3 — Additional file 3 Fig. S3. [file 12199_2021_973_MOESM3_ESM.png]

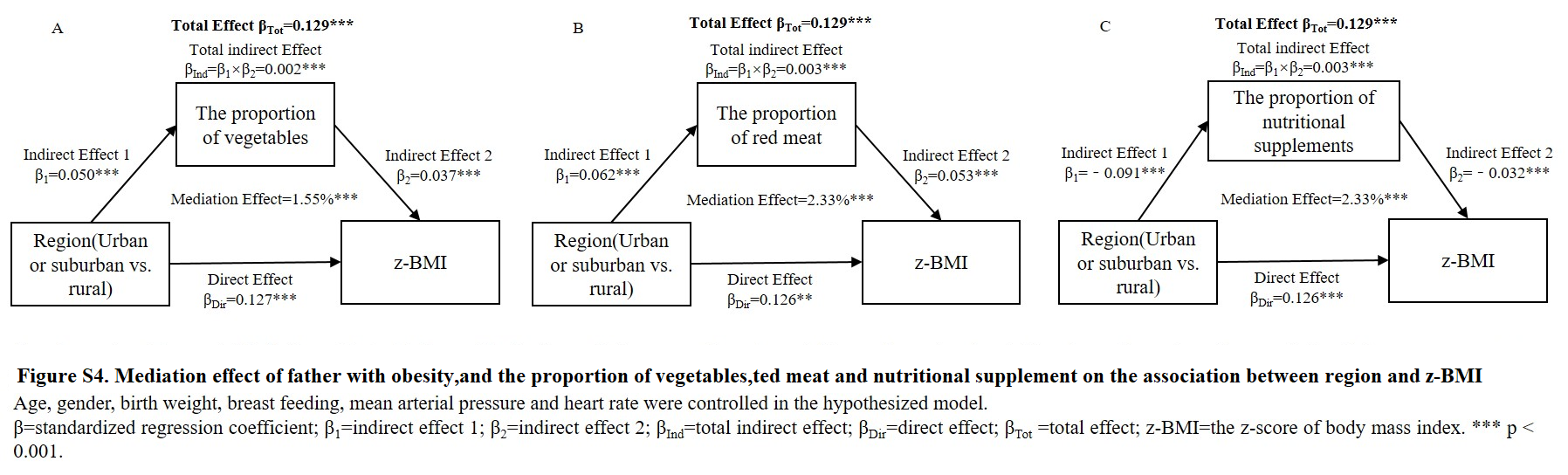

Supplement: Supplementary file 4 — Additional file 4 Fig. S4. [file 12199_2021_973_MOESM4_ESM.png]
