# Supplementary material for: The relationship between socioeconomic status and childhood overweight/obesity is linked through paternal obesity and dietary intake: a cross-sectional study in Chongqing, China
Source: Environ Health Prev Med. 2021 May 4;26:56. doi: 10.1186/s12199-021-00973-x (PMC8097861; doi:10.1186/s12199-021-00973-x)
Supplement: Supplementary file 5 — Additional file 5 Table S1. Dietary intakes. [file 12199_2021_973_MOESM5_ESM.docx]

| Table S1. Dietary intakes | | | | | |
| --- | --- | --- | --- | --- | --- |
| Variables | Total | Normal  (n=13272) | Overweight  (n=2272) | Obesity (n=1463) | P |
| **Mean ± STD, %** |  |  |  |  |  |
| ***Dietary intakes, %*** |  |  |  |  |  |
| Cereals and potatoes | 14.79±9.54 | 14.73±9.50 | 14.99±9.65 | 15.02±9.73 | 0.383 |
| Vegetables | 15.28±9.31 | 15.08±9.28 | 15.74±9.2 | 16.41±9.58 | <0.001 |
| Fruit | 12.80±8.78 | 12.88±8.81 | 12.62±8.65 | 12.36±8.67 | 0.096 |
| Red meat | 8.07±5.75 | 7.92±5.73 | 8.50±5.63 | 8.81±6.03 | <0.001 |
| Poultry | 3.65±3.84 | 3.66±3.81 | 3.52±3.68 | 3.85±4.32 | 0.082 |
| Fish | 2.31±3.22 | 2.34±3.25 | 2.21±3.11 | 2.17±3.11 | 0.108 |
| Eggs | 4.69±4.24 | 4.67±4.23 | 4.95±4.41 | 4.47±3.98 | 0.007 |
| Milk | 20.03±11.37 | 20.21±11.31 | 19.55±11.58 | 19.09±11.59 | 0.001 |
| Bean food | 3.64±4.28 | 3.66±4.28 | 3.63±4.26 | 3.51±4.35 | 0.518 |
| Nuts | 1.99±3.05 | 2.03±3.08 | 1.88±2.91 | 1.80±2.91 | 0.015 |
| Mushrooms and algae food | 1.79±2.95 | 1.78±2.90 | 1.89±3.33 | 1.73±2.71 | 0.274 |
| Oils | 5.67±4.46 | 5.67±4.50 | 5.67±4.34 | 5.68±4.27 | 0.999 |
| Pickle | 1.22±2.15 | 1.24±2.17 | 1.16±2.11 | 1.16±1.99 | 0.168 |
| Nutritional supplements | 1.04±3.14 | 1.10±3.21 | 0.83±2.80 | 0.79±3.00 | <0.001 |
| Beverage | 3.03±5.37 | 3.05±5.35 | 2.86±5.35 | 3.16±5.62 | 0.271 |
| **Median(P_25_,P_75_), g/day** |  |  |  |  |  |
| ***Dietary intakes, g/day*** |  |  |  |  |  |
| Cereals and potatoes | 150(100,250) | 150(100,250) | 178(100,250) | 178(100,250) | 0.030 |
| Vegetables | 150(100,250) | 150(100,250) | 200(100,250) | 200(100,300) | <0.001 |
| Fruit | 150(71,250) | 150(71,250) | 150(71,250) | 142(71,250) | 0.197 |
| Red meat | 100(50,150) | 100(50,150) | 100(50,150) | 100(50,150) | <0.001 |
| Poultry | 35(14,71) | 35(14,71) | 35(14,71) | 33(14,71) | 0.939 |
| Fish | 16(5,35) | 16(5,35) | 14(3,35) | 14(3,35) | 0.005 |
| Eggs | 50(18,100) | 50(16,100) | 50(21,100) | 50(16,100) | 0.003 |
| Milk | 250(160,260) | 250(180,268) | 250(150,285) | 250(142,250) | 0.023 |
| Bean food | 28(14,71) | 28(14,71) | 28(14,71) | 25(9,71) | 0.502 |
| Nuts | 13(2,35) | 13(2,35) | 11.67(2,35) | 8.33(1,33) | 0.011 |
| Mushrooms and algae food | 8(1,30) | 8.33(2,28) | 8.33(2,33) | 8.33(2,33) | 0.311 |
| Oils | 50(35,100) | 50(35,100) | 50(36,100) | 50(42,100) | 0.080 |
| Pickle | 7(0.5,16) | 7(0.5,18) | 6.(0.5,16) | 5(0.5,16) | 0.031 |
| Nutritional supplements | 0(0,5) | 0(0,6) | 0(0,2) | 0(0,1) | <0.001 |
| Beverage | 14(1,50) | 14(1,50) | 14(1,42) | 14(1,57) | 0.230 |
| **Mean ± STD, g/day** |  |  |  |  |  |
| ***Dietary intakes, g/day*** |  |  |  |  |  |
| Cereals and potatoes | 206.75±164.15 | 205.81±165.33 | 210.93±160.15 | 208.85±159.41 | 0.391 |
| Vegetables | 224.25±195.71 | 220.99±196.47 | 234.35±190.68 | 238.17±195.39 | 0.001 |
| Fruit | 189.67±179.89 | 190.11±179.76 | 190.37±177.80 | 184.67±184.22 | 0.574 |
| Red meat | 116.78±111.19 | 114.81±110.99 | 123.86±114.25 | 123.64±107.63 | <0.001 |
| Poultry | 58.90±83.14 | 58.85±82.79 | 57.19±79.55 | 61.95±91.16 | 0.283 |
| Fish | 37.43±74.77 | 38.08±76.17 | 36.48±72.50 | 33.02±64.56 | 0.055 |
| Eggs | 67.10±75.85 | 66.93±76.63 | 70.75±76.53 | 63.13±67.04 | 0.015 |
| Milk | 258.96±156.79 | 259.62±154.60 | 262.92±170.02 | 247.05±155.03 | 0.010 |
| Bean food | 57.88±90.71 | 57.85±90.22 | 59.18±93.13 | 56.17±91.51 | 0.643 |
| Nuts | 31.44±58.74 | 32.14±59.43 | 29.62±56.58 | 27.87±55.42 | 0.014 |
| Mushrooms and algae food | 28.74±62.44 | 28.83±62.94 | 29.46±66.71 | 26.90±50.03 | 0.490 |
| Oils | 73.58±61.13 | 73.15±60.92 | 76.81±65.24 | 72.53±56.24 | 0.039 |
| Pickle | 18.44±35.02 | 18.74±35.55 | 17.57±34.05 | 17.04±31.40 | 0.121 |
| Nutritional supplements | 18.51±64.34 | 19.46±65.48 | 15.85±59.28 | 13.97±60.93 | 0.002 |
| Beverage | 47.83±100.47 | 47.89±99.83 | 45.39±97.23 | 50.97±110.49 | 0.289 |
