# Supplementary material for: The relationship between socioeconomic status and childhood overweight/obesity is linked through paternal obesity and dietary intake: a cross-sectional study in Chongqing, China
Source: Environ Health Prev Med. 2021 May 4;26:56. doi: 10.1186/s12199-021-00973-x (PMC8097861; doi:10.1186/s12199-021-00973-x)
Supplement: Supplementary file 6 — Additional file 6 Table S2. The prevalence of overweight in adolescent by region and sex subgroups. [file 12199_2021_973_MOESM6_ESM.docx]

| **Table S2 The prevalence of overweight in adolescent by region and sex subgroups** | | | | | | | |
| --- | --- | --- | --- | --- | --- | --- | --- |
| **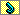Variables** | Urban | |  | Rural | | **χ^2^** | **P** |
|  | **Male** | **Female** |  | **Male** | **Female** |  |  |
| **Total** | 885(17.99%) | 527(11.75%) |  | 533(13.45%) | 327(8.98%) | 161.5 | <0.01 |
| Age, year |  |  |  |  |  |  |  |
| 6 | 93(14.83%) | 64(9.86%) |  | 44(11.49%) | 41(10.59%) | 15.31 | 0.02 |
| 7 | 163(16.60%) | 95(10.41%) |  | 85(13.24%) | 46(7.47%) |  |  |
| 8 | 149(18.44%) | 107(13.10%) |  | 78(12.02%) | 44(7.31%) |  |  |
| 9 | 155(19.42%) | 99(14.10%) |  | 74(12.19%) | 58(9.97%) |  |  |
| 10 | 147(18.77%) | 83(12.69%) |  | 98(13.59%) | 50(8.17%) |  |  |
| 11 | 139(19.94%) | 69(10.88%) |  | 105(15.04%) | 73(11.41%) |  |  |
| 12 | 39(17.41%) | 10(8.55%) |  | 49(18.70%) | 15(7.46%) |  |  |
| ***Socioeconomic index*** | |  |  |  |  |  |  |
| Father’s education, level, year^a^ | |  |  |  |  |  |  |
| ~9 | 303(16.34%) | 174(10.50%) |  | 317(13.04%) | 179(8.01%) | 18.29 | <0.01 |
| ~12 | 411(18.61%) | 271(13.17%) |  | 152(13.91%) | 103(9.95%) |  |  |
| ~15 | 139(20.47%) | 66(10.30%) |  | 57(16.86%) | 38(12.79%) |  |  |
| >15 | 11(25.58%) | 4(10.26%) |  | 2(14.29%) | 2(15.38%) |  |  |
| Father’s occupation^b^ | |  |  |  |  |  |  |
| Manager | 82(20.20%) | 49(12.76%) |  | 43(14.63%) | 32(12.80%) | 18.53 | <0.01 |
| Worker | 296(18.65%) | 182(12.60%) |  | 190(16.06%) | 105(9.55%) |  |  |
| Technicist/Researcher | 60(23.17%) | 26(9.96%) |  | 12(12.90%) | 10(10.20%) |  |  |
| Farmer | 211(15.91%) | 121(10.28%) |  | 187(12.14%) | 127(9.29%) |  |  |
| Others | 216(17.82%) | 139(12.20%) |  | 98(12.53%) | 52(6.66%) |  |  |
| Mother’s occupation^c^ | |  |  |  |  |  |  |
| Manager | 45(16.07%) | 33(13.31%) |  | 28(14.74%) | 20(11.49%) | 17.49 | <0.01 |
| Worker | 293(20.36%) | 173(12.34%) |  | 143(14.90%) | 91(9.98%) |  |  |
| Technicist/Researcher | 29(28.71%) | 8(8.79%) |  | 10(16.67%) | 5(10.64%) |  |  |
| Farmer | 279(17.49%) | 175(12.02%) |  | 147(14.07%) | 79(7.72%) |  |  |
| Others | 221(15.82%) | 130(10.67%) |  | 201(12.36%) | 129(9.09%) |  |  |
| Income, RMB^d^ |  |  |  |  |  |  |  |
| ~500 | 12(11.76%) | 8(8.42%) |  | 29(8.45%) | 22(7.26%) | 18.51 | <0.01 |
| ~1000 | 33(13.36%) | 27(12.05%) |  | 86(14.98%) | 40(6.83%) |  |  |
| ~2000 | 118(18.76%) | 60(10.40%) |  | 115(13.08%) | 62(7.55%) |  |  |
| >2000 | 565(18.33%) | 351(12.13%) |  | 281(13.97%) | 194(10.81%) |  |  |
| Live with grandparents^e^ | |  |  |  |  |  |  |
| No | 627(17.71%) | 370(11.23%) |  | 438(13.84%) | 270(9.30%) | 0.01 | 0.98 |
| Yes | 103(18.76%) | 77(15.01%) |  | 73(10.93%) | 50(8.09%) |  |  |
| People live with child^f^ | |  |  |  |  |  |  |
| 1 | 24(15.79%) | 18(12.08%) |  | 33(14.10%) | 13(6.67%) | 2.14 | 0.34 |
| 2~3 | 438(18.05%) | 260(12.34%) |  | 256(13.72%) | 155(9.38%) |  |  |
| 4 | 261(17.80%) | 163(10.85%) |  | 216(12.98%) | 142(8.83%) |  |  |
| Medical insurance^g^ | |  |  |  |  |  |  |
| No | 642(17.95%) | 397(12.09%) |  | 405(13.23%) | 255(8.97%) | 0.21 | 0.64 |
| Yes | 86(17.13%) | 49(9.72%) |  | 104(13.77%) | 63(9.66%) |  |  |
| ***Perinatal measures*** | |  |  |  |  |  |  |
| Gestational hypertension^h^ | |  |  |  |  |  |  |
| No | 707(17.94%) | 425(11.54%) |  | 490(13.61%) | 300(9.00%) | 1.85 | 0.17 |
| Yes | 12(22.22%) | 9(18.37%) |  | 7(10.77%) | 8(14.55%) |  |  |
| Birth weight, g^i^ |  |  |  |  |  |  |  |
| ~3000 | 159(16.99%) | 118(10.23%) |  | 96(10.46%) | 88(7.79%) | 13.77 | <0.01 |
| ~3600 | 350(18.38%) | 200(11.61%) |  | 226(14.18%) | 138(9.86%) |  |  |
| >3600 | 220(17.71%) | 128(13.85%) |  | 189(14.36%) | 94(9.57%) |  |  |
| Breast feeding, month^j^ | |  |  |  |  |  |  |
| 0~3 | 215(18.25%) | 132(11.50%) |  | 99(13.67%) | 61(8.70%) | 0.93 | 0.63 |
| 4~10 | 348(17.23%) | 246(11.96%) |  | 248(14.27%) | 152(9.54%) |  |  |
| >10 | 159(18.95%) | 64(11.23%) |  | 151(11.84%) | 102(8.79%) |  |  |
| Father with obesity^k^ | |  |  |  |  |  |  |
| No | 574(17.01%) | 339(10.76%) |  | 400(12.46%) | 249(8.51%) | 40.08 | <0.01 |
| Yes | 153(22.34%) | 101(16.29%) |  | 99(17.16%) | 63(11.60%) |  |  |
| Mother with obesity^l^ | |  |  |  |  |  |  |
| No | 651(17.58%) | 368(10.82%) |  | 433(12.69%) | 257(8.36%) | 45.06 | <0.01 |
| Yes | 79(22.07%) | 69(18.75%) |  | 70(18.62%) | 56(14.47%) |  |  |

^a^A total of 373 subjects having missing values in mother’s education level.

^b^A total of 323 subjects having missing value in father’s occupation.

^c^A total of 324 subjects having missing value in mother’s occupation.

^d^A total of 1845subjects having missing values in parents’ income.

^e^A total of 1754subjects having missing values in live with grandparents.

^f^A total of 1984 subjects having missing values in people live with.

^g^A total of 1827 subjects having missing values in medical insurance.

^g^A total of 1827 subjects having missing values in medical insurance.

^i^A total of 1786 subjects having missing values in birth weight.

^j^A total of 2004 subjects having missing values in breastfeeding.

^k^A total of 1920 subjects having missing values in father with obesity.

^l^A total of 1926 subjects having missing values in mother with obesity.
