# Supplementary material for: The relationship between socioeconomic status and childhood overweight/obesity is linked through paternal obesity and dietary intake: a cross-sectional study in Chongqing, China
Source: Environ Health Prev Med. 2021 May 4;26:56. doi: 10.1186/s12199-021-00973-x (PMC8097861; doi:10.1186/s12199-021-00973-x)
Supplement: Supplementary file 7 — Additional file 7 Table S3. The prevalence of obesity in adolescent by region and sex subgroups. [file 12199_2021_973_MOESM7_ESM.docx]

| **Table S3 The prevalence of obesity in adolescent by region and sex subgroups** | | | | | | | |
| --- | --- | --- | --- | --- | --- | --- | --- |
| **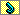Variables** | Urban | |  | Rural | | **χ^2^** | P |
|  | **Male** | **Female** |  | **Male** | **Female** |  |  |
| total | 677(13.76%) | 216(4.81%) |  | 418(10.55%) | 152(4.18%) | 358.3 | <0.01 |
| Age, year |  |  |  |  |  |  |  |
| 6 | 95(15.15%) | 42(6.47%) |  | 45(11.75%) | 18(4.65%) | 16.2 | 0.01 |
| 7 | 151(15.38%) | 46(5.04%) |  | 60(9.35%) | 27(4.38%) |  |  |
| 8 | 116(14.36%) | 40(4.90%) |  | 73(11.25%) | 27(4.49%) |  |  |
| 9 | 104(13.03%) | 25(3.56%) |  | 69(11.37%) | 29(4.98%) |  |  |
| 10 | 114(14.56%) | 27(4.13%) |  | 70(9.71%) | 25(4.08%) |  |  |
| 11 | 77(11.05%) | 32(5.05%) |  | 80(11.46%) | 23(3.59%) |  |  |
| 12 | 20(8.93%) | 4(3.42%) |  | 21(8.02%) | 3(1.49%) |  |  |
| ***Socioeconomic index*** |  |  |  |  |  |  |  |
| Father’s education, level, year^a^ |  |  |  |  |  |  |  |
| ~9 | 217(11.70%) | 86(5.19%) |  | 218(8.97%) | 91(4.07%) | 18.41 | <0.01 |
| ~12 | 319(14.45%) | 93(4.52%) |  | 147(13.45%) | 46(4.44%) |  |  |
| ~15 | 117(17.23%) | 30(4.68%) |  | 39(11.54%) | 11(3.70%) |  |  |
| >15 | 7(16.28%) | 1(2.56%) |  | 2(14.29%) | 3(23.08%) |  |  |
| Father’s occupation^b^ |  |  |  |  |  |  |  |
| Manager | 53(13.05%) | 20(5.21%) |  | 37(12.59%) | 7(2.80%) | 9.12 | 0.06 |
| Worker | 245(15.44%) | 69(4.78%) |  | 129(10.90%) | 47(4.27%) |  |  |
| Technicist/Researcher | 35(13.51%) | 11(4.21%) |  | 14(15.05%) | 7(7.14%) |  |  |
| Farmer | 164(12.37%) | 57(4.84%) |  | 136(8.83%) | 53(3.88%) |  |  |
| Others | 165(13.61%) | 53(4.65%) |  | 92(11.76%) | 38(4.87%) |  |  |
| Mother’s occupation^c^ |  |  |  |  |  |  |  |
| Manager | 38(13.57%) | 12(4.84%) |  | 27(14.21%) | 6(3.45%) | 7.48 | 0.11 |
| Worker | 201(13.97%) | 63(4.49%) |  | 99(10.31%) | 35(3.84%) |  |  |
| Technicist/Researcher | 16(15.84%) | 7(7.69%) |  | 9(15.00%) | 4(8.51%) |  |  |
| Farmer | 232(14.55%) | 67(4.60%) |  | 112(10.72%) | 47(4.59%) |  |  |
| Others | 173(12.38%) | 61(5.01%) |  | 157(9.66%) | 57(4.02%) |  |  |
| Income, RMB^d^ |  |  |  |  |  |  |  |
| ~500 | 12(11.76%) | 5(5.26%) |  | 23(6.71%) | 13(4.29%) | 9.37 | 0.02 |
| ~1000 | 31(12.55%) | 9(4.02%) |  | 55(9.58%) | 22(3.75%) |  |  |
| ~2000 | 80(12.72%) | 33(5.72%) |  | 95(10.81%) | 24(2.92%) |  |  |
| >2000 | 453(14.70%) | 141(4.87%) |  | 227(11.29%) | 91(5.07%) |  |  |
| Live with grandparents^e^ |  |  |  |  |  |  |  |
| No | 495(13.98%) | 169(5.13%) |  | 338(10.68%) | 125(4.31%) | 0.16 | 0.69 |
| Yes | 83(15.12%) | 19(3.70%) |  | 67(10.03%) | 26(4.21%) |  |  |
| People live with child^f^ |  |  |  |  |  |  |  |
| 1 | 23(15.13%) | 13(8.72%) |  | 26(11.11%) | 6(3.08%) | 2.32 | 0.31 |
| 2~3 | 347(14.30%) | 99(4.70%) |  | 207(11.09%) | 75(4.54%) |  |  |
| 4 | 200(13.64%) | 75(4.99%) |  | 160(9.62%) | 65(4.04%) |  |  |
| Medical insurance^g^ |  |  |  |  |  |  |  |
| No | 508(14.20%) | 167(5.09%) |  | 317(10.35%) | 120(4.22%) | 0.01 | 0.96 |
| Yes | 69(13.75%) | 21(4.17%) |  | 85(11.26%) | 29(4.45%) |  |  |
| ***Perinatal measures*** |  |  |  |  |  |  |  |
| Gestational hypertension^h^ |  |  |  |  |  |  |  |
| No | 559(14.18%) | 179(4.86%) |  | 373(10.36%) | 142(4.26%) | 0.01 | 0.98 |
| Yes | 5(9.26%) | 5(10.20%) |  | 8(12.31%) | 1(1.82%) |  |  |
| Birth weight, g^i^ |  |  |  |  |  |  |  |
| ~3000 | 102(10.90%) | 49(4.25%) |  | 81(8.82%) | 40(3.54%) | 20.95 | <0.01 |
| ~3600 | 267(14.02%) | 91(5.28%) |  | 172(10.79%) | 64(4.57%) |  |  |
| >3600 | 208(16.75%) | 49(5.30%) |  | 151(11.47%) | 46(4.68%) |  |  |
| Breast feeding, month^j^ |  |  |  |  |  |  |  |
| 0~3 | 178(15.11%) | 61(5.31%) |  | 88(12.15%) | 27(3.85%) | 2.41 | 0.3 |
| 4~10 | 286(14.16%) | 105(5.11%) |  | 175(10.07%) | 59(3.70%) |  |  |
| >10 | 109(12.99%) | 22(3.86%) |  | 134(10.51%) | 61(5.26%) |  |  |
| Father with obesity^k^ |  |  |  |  |  |  |  |
| No | 431(12.77%) | 120(3.81%) |  | 292(9.10%) | 105(3.59%) | 145.2 | <0.01 |
| Yes | 142(20.73%) | 67(10.81%) |  | 110(19.06%) | 45(8.29%) |  |  |
| Mother with obesity^l^ |  |  |  |  |  |  |  |
| No | 478(12.91%) | 150(4.41%) |  | 336(9.85%) | 118(3.84%) | 102.4 | <0.01 |
| Yes | 95(26.54%) | 36(9.78%) |  | 66(17.55%) | 30(7.75%) |  |  |

^a^A total of 373 subjects having missing values in mother’s education level.

^b^A total of 323 subjects having missing value in father’s occupation.

^c^A total of 324 subjects having missing value in mother’s occupation.

^d^A total of 1845subjects having missing values in parents’ income.

^e^A total of 1754subjects having missing values in live with grandparents.

^f^A total of 1984 subjects having missing values in people live with.

^g^A total of 1827 subjects having missing values in medical insurance.

^g^A total of 1827 subjects having missing values in medical insurance.

^i^A total of 1786 subjects having missing values in birth weight.

^j^A total of 2004 subjects having missing values in breastfeeding.

^k^A total of 1920 subjects having missing values in father with obesity.

^l^A total of 1926 subjects having missing values in mother with obesity.
