# Supplementary material for: The relationship between socioeconomic status and childhood overweight/obesity is linked through paternal obesity and dietary intake: a cross-sectional study in Chongqing, China
Source: Environ Health Prev Med. 2021 May 4;26:56. doi: 10.1186/s12199-021-00973-x (PMC8097861; doi:10.1186/s12199-021-00973-x)
Supplement: Supplementary file 8 — Additional file 8 Table S4. Results of univariate logistic model of the risk factors for childhood overweight/obesity. [file 12199_2021_973_MOESM8_ESM.docx]

| Table S4. Results of Univariate Logistic Model of the Risk Factors for Childhood Overweight/Obesity | | | | | | | |
| --- | --- | --- | --- | --- | --- | --- | --- |
| Variables^a^ | Overweight vs. Normal | | |  | Obesity vs. Normal | | |
|  | β | P | OR(95%CI) |  | β | P | OR(95%CI) |
| ***General characteristics*** |  |  |  |  |  |  |  |
| Region( Urban or suburb vs. rural) | 0.373 | <0.001 | 1.453(1.325,1.593) |  | 0.301 | <0.001 | 1.352(1.208,1.512) |
| Father’s education, ref. ≤9 year |  |  |  |  |  |  |  |
| ~12 | 0.287 | <0.001 | 1.332(1.208,1.469) |  | 0.302 | <0.001 | 1.352(1.199,1.524) |
| ~15 | 0.354 | <0.001 | 1.425(1.236,1.643) |  | 0.374 | <0.001 | 1.453(1.222,1.727) |
| >15 | 0.536 | 0.040 | 1.708(1.026,2.846) |  | 0.613 | 0.046 | 1.846(1.011,3.373) |
| **P for trend** |  | <0.001 |  |  |  | <0.001 |  |
| Father’s occupation, ref. worker |  |  |  |  |  |  |  |
| Manager | 0.062 | 0.475 | 1.064(0.898,1.26) |  | -0.041 | 0.708 | 0.96(0.773,1.191) |
| Technicist/Researcher | 0.077 | 0.495 | 1.08(0.865,1.349) |  | 0.048 | 0.730 | 1.05(0.798,1.381) |
| Farmer | -0.256 | <0.001 | 0.774(0.691,0.868) |  | -0.285 | <0.001 | 0.752(0.654,0.865) |
| Others | -0.138 | 0.027 | 0.871(0.771,0.984) |  | -0.059 | 0.432 | 0.943(0.814,1.092) |
| Mother’s occupation, ref. worker |  |  |  |  |  |  |  |
| Manager | -0.060 | 0.570 | 0.942(0.765,1.159) |  | 0.085 | 0.510 | 1.089(0.845,1.403) |
| Technicist/Researcher | 0.242 | 0.133 | 1.274(0.929,1.749) |  | 0.397 | 0.037 | 1.488(1.024,2.163) |
| Farmer | -0.130 | 0.028 | 0.878(0.783,0.986) |  | 0.033 | 0.648 | 1.034(0.896,1.193) |
| Others | -0.268 | <0.001 | 0.765(0.682,0.858) |  | -0.151 | 0.039 | 0.859(0.744,0.992) |
| Income, ref.≤500 RMB |  |  |  |  |  |  |  |
| ~1000 | 0.365 | 0.013 | 1.441(1.079,1.924) |  | 0.218 | 0.210 | 1.243(0.885,1.746) |
| ~2000 | 0.448 | 0.001 | 1.565(1.195,2.048) |  | 0.329 | 0.039 | 1.39(1.017,1.9) |
| >2000 | 0.647 | <0.001 | 1.909(1.485,2.455) |  | 0.527 | <0.001 | 1.695(1.268,2.264) |
| **P for trend** |  | <0.001 |  |  |  | <0.001 |  |
| Live with grandparents (Yes vs. No) | -0.030 | 0.657 | 0.97(0.85,1.108) |  | -0.069 | 0.402 | 0.933(0.794,1.097) |
| People live with child, ref. 1 |  |  |  |  |  |  |  |
| 2~3 | 0.155 | 0.195 | 1.168(0.924,1.476) |  | -0.029 | 0.830 | 0.971(0.744,1.267) |
| 4 | 0.052 | 0.669 | 1.053(0.83,1.336) |  | -0.162 | 0.243 | 0.851(0.649,1.116) |
| **P for trend** |  | 0.227 |  |  |  | 0.032 |  |
| Medical insurance (Yes vs. No) | -0.076 | 0.264 | 0.927(0.812,1.059) |  | -0.048 | 0.551 | 0.953(0.813,1.117) |
| ***Perinatal measures*** |  |  |  |  |  |  |  |
| Gestational hypertension(Yes vs. No) | 0.234 | 0.210 | 1.263(0.877,1.821) |  | 0.011 | 0.965 | 1.011(0.623,1.639) |
| Birth weight, ref. ~3000g |  |  |  |  |  |  |  |
| 3000~3600 | 0.239 | <0.001 | 1.27(1.125,1.433) |  | 0.298 | <0.001 | 1.347(1.158,1.568) |
| >3600 | 0.253 | <0.001 | 1.288(1.13,1.467) |  | 0.422 | <0.001 | 1.525(1.3,1.79) |
| **P for trend** |  | <0.001 |  |  |  | <0.001 |  |
| Breast feeding, ref. 0~3 month |  |  |  |  |  |  |  |
| 4~10 | -0.025 | 0.672 | 0.975(0.868,1.096) |  | -0.126 | 0.078 | 0.882(0.767,1.014) |
| >10 | -0.149 | 0.032 | 0.862(0.752,0.987) |  | -0.169 | 0.040 | 0.845(0.719,0.993) |
| **P for trend** |  | 0.032 |  |  |  | 0.040 |  |
| Father with obesity (Yes vs. No) | 0.513 | <0.001 | 1.671(1.481,1.885) |  | 0.892 | <0.001 | 2.44(2.134,2.79) |
| Mother with obesity (Yes vs. No) | 0.595 | <0.001 | 1.813(1.569,2.094) |  | 0.908 | <0.001 | 2.481(2.112,2.913) |
| ***Anthropometric measures*** |  |  |  |  |  |  |  |
| Heart rate, n/min | 0.001 | 0.441 | 1.001(0.998,1.005) |  | 0.011 | <0.001 | 1.011(1.007,1.016) |
| SBP, mmHg | 0.058 | <0.001 | 1.060(1.055,1.065) |  | 0.090 | <0.001 | 1.094(1.088,1.100) |
| DBP, mmHg | 0.036 | <0.001 | 1.037(1.031,1.043) |  | 0.066 | <0.001 | 1.068(1.061,1.076) |
| MAP, mmHg | 0.056 | <0.001 | 1.057(1.051,1.064) |  | 0.092 | <0.001 | 1.097(1.089,1.105) |
| ***Dietary intaking, %*** |  |  |  |  |  |  |  |
| Cereals and potatoes | 0.002 | 0.389 | 1.002(0.997,1.008) |  | 0.003 | 0.427 | 1.003(0.996,1.009) |
| Vegetables | 0.008 | 0.002 | 1.009(1.003,1.014) |  | 0.018 | <0.001 | 1.018(1.012,1.024) |
| Fruit | -0.002 | 0.559 | 0.998(0.993,1.004) |  | -0.004 | 0.303 | 0.996(0.989,1.003) |
| Red meat | 0.016 | <0.001 | 1.016(1.007,1.024) |  | 0.024 | <0.001 | 1.024(1.014,1.034) |
| Poultry | -0.011 | 0.100 | 0.989(0.975,1.002) |  | 0.009 | 0.228 | 1.009(0.994,1.025) |
| Fish | -0.012 | 0.167 | 0.988(0.972,1.005) |  | -0.017 | 0.108 | 0.983(0.963,1.004) |
| Eggs | 0.016 | 0.006 | 1.016(1.004,1.028) |  | -0.013 | 0.097 | 0.987(0.972,1.002) |
| Milk | -0.005 | 0.019 | 0.995(0.990,0.999) |  | -0.011 | <0.001 | 0.989(0.984,0.995) |
| Bean food | -0.003 | 0.673 | 0.997(0.986,1.009) |  | -0.010 | 0.193 | 0.990(0.976,1.005) |
| Nuts | -0.014 | 0.114 | 0.986(0.969,1.003) |  | -0.024 | 0.033 | 0.976(0.955,0.998) |
| Mushrooms and algae food | 0.013 | 0.117 | 1.013(0.997,1.029) |  | -0.003 | 0.764 | 0.997(0.976,1.018) |
| Oils | 0.001 | 0.846 | 1.001(0.990,1.013) |  | 0.001 | 0.895 | 1.001(0.987,1.015) |
| Pickle | -0.018 | 0.145 | 0.982(0.958,1.006) |  | -0.015 | 0.307 | 0.985(0.956,1.014) |
| Nutritional supplements | -0.032 | 0.001 | 0.969(0.950,0.987) |  | -0.041 | 0.001 | 0.960(0.937,0.983) |
| Beverage | -0.011 | 0.031 | 0.989(0.979,0.999) |  | -0.001 | 0.882 | 0.999(0.988,1.010) |
| ^a^Adjusted age and gender. |  |  |  |  |  |  |  |
