# Supplementary material for: The relationship between socioeconomic status and childhood overweight/obesity is linked through paternal obesity and dietary intake: a cross-sectional study in Chongqing, China
Source: Environ Health Prev Med. 2021 May 4;26:56. doi: 10.1186/s12199-021-00973-x (PMC8097861; doi:10.1186/s12199-021-00973-x)
Supplement: Supplementary file 9 — Additional file 9 Table S5. Model of SES, perinatal and anthropometric for childhood overweight and Obesity. [file 12199_2021_973_MOESM9_ESM.docx]

| Table S5. Model of SES, Perinatal and Anthropometric for childhood Overweight and Obesity | | | | | | | |  |
| --- | --- | --- | --- | --- | --- | --- | --- | --- |
| Variables | Overweight vs. Normal | | |  | Obesity vs. Normal | | | R^2^ |
|  | β | P | OR(95%CI) |  | β | P | OR(95%CI) |  |
| ***SES model^a^*** |  |  |  |  |  |  |  | 1.38% |
| Region( Urban or suburb vs. rural) | 0.241 | <0.001 | 1.272(1.149,1.409) |  | 0.235 | <0.001 | 1.265(1.118,1.432) |  |
| Father’s education, ref. ≤9 year |  |  |  |  |  |  |  |  |
| ~12 | 0.164 | 0.005 | 1.178(1.052,1.32) |  | 0.226 | 0.001 | 1.254(1.093,1.439) |  |
| ~15 | 0.159 | 0.080 | 1.172(0.981,1.401) |  | 0.308 | 0.004 | 1.361(1.101,1.681) |  |
| >15 | 0.320 | 0.262 | 1.377(0.788,2.408) |  | 0.436 | 0.192 | 1.546(0.804,2.973) |  |
| Father’s occupation, ref. worker |  |  |  |  |  |  |  |  |
| Manager | -0.015 | 0.882 | 0.985(0.803,1.208) |  | -0.216 | 0.098 | 0.806(0.624,1.04) |  |
| Technicist/Researcher | -0.101 | 0.438 | 0.904(0.7,1.167) |  | -0.232 | 0.145 | 0.793(0.58,1.083) |  |
| Farmer | -0.152 | 0.066 | 0.859(0.73,1.01) |  | -0.253 | 0.011 | 0.777(0.639,0.944) |  |
| Others | -0.134 | 0.097 | 0.874(0.746,1.025) |  | -0.139 | 0.146 | 0.87(0.721,1.05) |  |
| Mother’s occupation, ref. worker |  |  |  |  |  |  |  |  |
| Manager | -0.116 | 0.355 | 0.891(0.697,1.138) |  | 0.073 | 0.631 | 1.076(0.799,1.449) |  |
| Technicist/Researcher | 0.266 | 0.141 | 1.305(0.916,1.86) |  | 0.530 | 0.011 | 1.699(1.127,2.56) |  |
| Farmer | -0.043 | 0.574 | 0.958(0.824,1.113) |  | 0.167 | 0.072 | 1.182(0.985,1.417) |  |
| Others | -0.014 | 0.867 | 0.986(0.835,1.164) |  | 0.213 | 0.038 | 1.238(1.012,1.514) |  |
| Income, ref.≤500 RMB |  |  |  |  |  |  |  |  |
| ~1000 | 0.355 | 0.018 | 1.426(1.063,1.913) |  | 0.191 | 0.277 | 1.211(0.857,1.71) |  |
| ~2000 | 0.372 | 0.008 | 1.451(1.102,1.91) |  | 0.234 | 0.152 | 1.264(0.917,1.741) |  |
| >2000 | 0.472 | <0.001 | 1.603(1.234,2.082) |  | 0.344 | 0.026 | 1.41(1.042,1.909) |  |
| ***Perinatal measures model^b^*** |  |  |  |  |  |  |  | 2.90% |
| Birth weight, ref. ~3000g |  |  |  |  |  |  |  |  |
| 3000~3600 | 0.263 | <0.001 | 1.301(1.152,1.47) |  | 0.374 | <0.001 | 1.453(1.248,1.692) |  |
| >3600 | 0.301 | <0.001 | 1.352(1.184,1.543) |  | 0.497 | <0.001 | 1.643(1.398,1.932) |  |
| Breast feeding, ref. 0~3 month |  |  |  |  |  |  |  |  |
| 4~10 | -0.042 | 0.484 | 0.959(0.852,1.079) |  | -0.154 | 0.032 | 0.858(0.745,0.987) |  |
| >10 | -0.132 | 0.058 | 0.876(0.764,1.005) |  | -0.175 | 0.033 | 0.839(0.714,0.986) |  |
| Father with obesity (Yes vs. No) | 0.412 | <0.001 | 1.509(1.333,1.71) |  | 0.752 | <0.001 | 2.122(1.849,2.434) |  |
| Mother with obesity (Yes vs. No) | 0.449 | <0.001 | 1.567(1.349,1.819) |  | 0.608 | <0.001 | 1.837(1.557,2.168) |  |
| ***Anthropometric measures model^c^*** |  |  |  |  |  |  |  | 0.70% |
| MAP, mmHg | 0.056 | <0.001 | 1.057(1.051,1.064) |  | 0.092 | <0.001 | 1.097(1.089,1.105) |  |
| ***Dietary intaking model^d^*** |  |  |  |  |  |  |  | 0.99% |
| Vegetables, % | 0.007 | 0.02 | 1.007(1.001,1.013) |  | 0.012 | 0.001 | 1.012(1.005,1.019) |  |
| Red meat, % | 0.017 | <0.001 | 1.017(1.008,1.026) |  | 0.024 | <0.001 | 1.024(1.014,1.034) |  |
| Eggs, % | 0.017 | 0.004 | 1.017(1.005,1.029) |  | -0.008 | 0.292 | 0.992(0.977,1.007) |  |
| Milk, % | -0.003 | 0.237 | 0.997(0.992,1.002) |  | -0.006 | 0.057 | 0.994(0.988,1.000) |  |
| Nuts, % | -0.012 | 0.185 | 0.988(0.970,1.006) |  | -0.020 | 0.088 | 0.981(0.959,1.003) |  |
| Nutritional supplements, % | -0.028 | 0.005 | 0.973(0.954,0.992) |  | -0.033 | 0.008 | 0.968(0.945,0.992) |  |
| MAP, mean arterial pressure.  ^a^A total of 14784 subjects being included in SES model.  ^b^A total of 14741 subjects being included in perinatal measures model.  ^c^A total of 15136 subjects being included in anthropometric measures model.  ^d^A total of 13561 subjects being included in dietary intaking model. | | | | | | | |  |
