# Supplementary material for: The relationship between socioeconomic status and childhood overweight/obesity is linked through paternal obesity and dietary intake: a cross-sectional study in Chongqing, China
Source: Environ Health Prev Med. 2021 May 4;26:56. doi: 10.1186/s12199-021-00973-x (PMC8097861; doi:10.1186/s12199-021-00973-x)
Supplement: Supplementary file 10 — Additional file 10 Table S6. Mediation model fit indexes. [file 12199_2021_973_MOESM10_ESM.docx]

| Table S6. Mediation model fit indexes. | | | | | | | |
| --- | --- | --- | --- | --- | --- | --- | --- |
| Mediation model^a^ | | | χ2/df | GFI≥0.9 | CFI≥0.9 | TLI≥0.9 | RMSEA<0.05 |
| Independent variable | Mediator variable | Dependent variable |  |  |  |  |  |
| Income | Mother with obesity | z-BMI | 26.296 | 0.989 | 0.857 | 0.786 | 0.044 |
| Income | Father with obesity | z-BMI | 10.008 | 0.997 | 0.957 | 0.923 | 0.027 |
| Income | The proportion of vegetables | z-BMI | 21.899 | 0.992 | 0.895 | 0.820 | 0.040 |
| Income | The proportion of red meat | z-BMI | 11.254 | 0.996 | 0.951 | 0.912 | 0.028 |
| Income | The proportion of nutritional supplements | z-BMI | 9.871 | 0.997 | 0.957 | 0.923 | 0.026 |
| Father education | Mother with obesity | z-BMI | 33.210 | 0.987 | 0.840 | 0.738 | 0.050 |
| Father education | Father with obesity | z-BMI | 12.767 | 0.996 | 0.947 | 0.904 | 0.030 |
| Father education | The proportion of vegetables | z-BMI | 20.920 | 0.992 | 0.904 | 0.835 | 0.039 |
| Father education | The proportion of red meat | z-BMI | 12.641 | 0.996 | 0.949 | 0.906 | 0.030 |
| Father education | The proportion of nutritional supplements | z-BMI | 11.152 | 0.996 | 0.956 | 0.916 | 0.028 |
| Region(Urban or suburban vs. rural) | Mother with obesity | z-BMI | 39.926 | 0.985 | 0.828 | 0.705 | 0.055 |
| Region(Urban or suburban vs. rural) | Father with obesity | z-BMI | 25.188 | 0.991 | 0.897 | 0.815 | 0.043 |
| Region(Urban or suburban vs. rural) | The proportion of vegetables | z-BMI | 11.157 | 0.997 | 0.965 | 0.922 | 0.028 |
| Region(Urban or suburban vs. rural) | The proportion of red meat | z-BMI | 11.128 | 0.997 | 0.966 | 0.922 | 0.028 |
| Region(Urban or suburban vs. rural) | The proportion of nutritional supplements | z-BMI | 9.138 | 0.993 | 0.972 | 0.937 | 0.025 |
| ^a^Age, gender, birth weight, breast feeding, mean arterial pressure and heart rate were controlled in the hypothesized model.  df= degrees of freedom; GFI = goodness-of-fit index ; CFI = confirmatory fit index; TLI = Tucker–Lewis index; RMSEA = root mean square error of approximation; z-BMI=the z-score of body mass index. | | | | | | | |
